# Systems Biology and Chemoinformatics-Based Strategies to Explore the Biological Mechanism of Fugui Wenyang Decoction in Treating Vascular Dementia Rats

**DOI:** 10.1155/2021/6693955

**Published:** 2021-10-07

**Authors:** Kailin Yang, Liuting Zeng, Anqi Ge, Chuandong Cao, Haiyan Zhang, Tingting Bao, Yaqiao Yi, Jinwen Ge

**Affiliations:** ^1^Hunan University of Chinese Medicine, Changsha, Hunan Province, China; ^2^The First Affiliated Hospital of Hunan University of Chinese Medicine, Changsha, Hunan Province, China; ^3^Beijing University of Chinese Medicine, Beijing, China

## Abstract

**Objective:**

To explore the biological mechanism of Fugui Wenyang Decoction (FGWYD) in treating vascular dementia (VD) rats based on systems pharmacology, proteomics, and a multidirectional pharmacology integration strategy.

**Methods:**

Chemoinformatics was utilized to construct and analyze the FGWYD-VD protein-protein interaction (PPI) network. Then, the total protein in the brain tissue of the infarcted side of the rat was extracted for protein identification, pattern identification, and protein quantitative analysis. The differentially expressed proteins are analyzed by bioinformatics. Finally, the important proteins in the oxidative stress-related biological process proteins and indicators were detected through experimental pharmacology to verify the findings of systems biology and chemoinformatics.

**Results:**

There were a total of 73 FGWYD components with 245 FGWYD and 145 VD genes. The results of GO enrichment analysis and pathway enrichment analysis showed that MBHD may regulate the inflammation module, oxidative stress, the synaptic plasticity regulation module, and the neuronal apoptosis section module. Compared with the sham operation group, there were 23 upregulated proteins and 17 downregulated proteins in the model group (*P* < 0.05). Compared with the model group, there were 16 upregulated proteins and 10 downregulated proteins in the FGWYD group (*P* < 0.05). Bioinformatics analysis shows that those proteins were closely related to processes such as inflammation, oxidative stress, neuronal apoptosis, neuronal growth and differentiation, signaling pathways, and transcriptional regulation. Multidirectional pharmacology further verified the neuroprotective mechanism of the Nrf2/HO-1 pathway in FGWYD treatment of VD.

**Conclusion:**

The mechanism of FGWYD in the treatment of VD may be related to inflammation, oxidative stress, angiogenesis, and neuronal apoptosis.

## 1. Introduction

Vascular dementia (VD) refers to a clinical syndrome characterized by a decline in cognitive functions such as learning ability, memory function, computing power, and orientation caused by various cerebrovascular accidents [1, 2]. The incidence of VD accounts for about 20% of all dementia types [3]. Epidemiological studies have shown that with the extension of lifespan and the increase in the incidence of cerebrovascular diseases, the population affected by VD is also increasing year by year. It is predicted that by 2040, the number of people with dementia worldwide will reach 81.1 million [4]. VD is divided into four categories according to the causes and clinical manifestations [5]: (1) mild vascular cognitive impairment, (2) mixed dementia, (3) dementia caused by vascular disease, and (4) postapoplectic dementia. The clinical manifestations of VD are related to the location, size, and number of infarctions. The symptoms of patients can be roughly divided into memory loss, abnormal executive function, daily activity ability, and mental and behavioral abnormalities. The pathogenesis of VD has not been fully elucidated, and it may be closely related to excitatory amino acid toxicity, nerve cell apoptosis, free radical damage, and inflammatory response [6–8]. Neuroimaging and pathological studies have confirmed that due to cerebral vascular obstruction or traumatic lesions, insufficient cerebral blood flow perfusion is the main factor for cognitive dysfunction caused by VD [8].

At present, the drugs used in the treatment of VD are roughly classified into the following categories [9–11]: (1) acetylcholinesterase inhibitors, such as donepezil and galantamine; (2) brain circulation- and brain metabolism-promoting drugs, such as piracetam, pyrithione, and ergot alkaloid; and (3) neuroprotective drugs, such as calcium antagonists and antioxidants. Clinically, VD often requires a combination of these drugs to promote VD management. Traditional Chinese Medicine (TCM) has played an active role in VD management. TCM has a significant impact on the clinical prevention and treatment of VD because of its synergistic combination and fewer side effects. For single-target chemical drugs, the application of the compound effectively compensates for the defects of Western medicine in clinical application, and has great advantages [12, 13].

**Figure 1:**
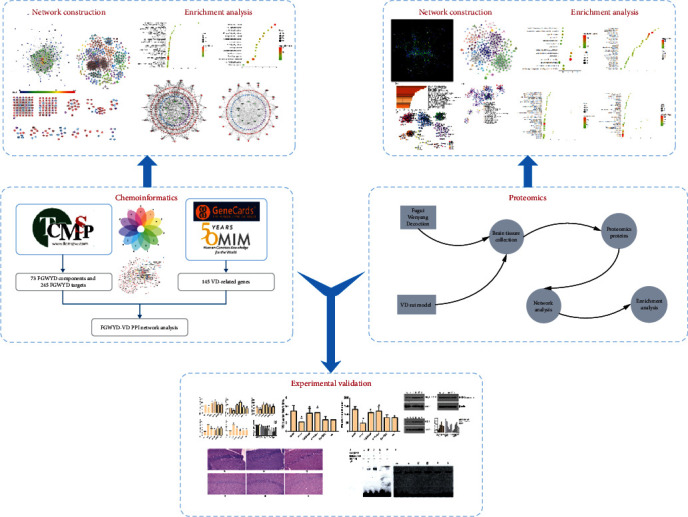
Research ideas.

Our team created Fugui Wenyang Decoction (FGWYD) based on a large number of clinical practices based on the theory of TCM. FGWYD is composed of *Aconiti Lateralis Radix Praeparata* 15 g, *Zingiberis Rhizoma* 15 g, *Acoritataninowii Rhizoma* 15 g, *Epimrdii Herba* 15 g, *Cinnamomi Ramulus* 15 g, *Morindae Officinalis Radix* 15 g, *Arum Ternatum Thunb.* 15 g, *Panax Ginseng C. A. Mey.* 15 g, *Panax Notoginseng* (*Burk.*) *F. H. Chen Ex C. Chow* 15 g, and *Radix Rhei Et Rhizome* 6 g. The multicenter clinical randomized controlled trial confirmed that the FGWYD group was superior to the monadipine tablet group in terms of the recovery of daily living ability and brain nerve mediators (such as norepinephrine, dopamine, and etocholine) [14, 15]. Our previous research found that FGWYD can inhibit the overexpression of CDK5 and thereby inhibit the hyperphosphorylation of Tau protein to reduce brain nerve fiber tangles, significantly improving the cognitive ability of SAMP8 mice [16]. However, previous studies have mainly focused on the study of a single signaling pathway, and there is a lack of systematic research. More importantly, due to the characteristics of TCM's “multiple compounds and multiple targets,” it is difficult to explore its overall efficacy. Therefore, this study will integrate network pharmacology and proteomics strategies to comprehensively analyze the mechanism of FGWYD regulating VD biological network. The idea of this research is shown in Figure 1.

## 2. Materials and Methods

### 2.1. FGWYD Potential Component Collection

TCMSP (https://tcmspw.com/tcmsp.php) [17] was used to search for keywords such as “*Aconiti Lateralis Radix Praeparata*” and “*Zingiberis Rhizoma*” to collect the chemical components in FGWYD and the pharmacokinetic parameters of each chemical component. Oral bioavailability (OB) ≥ 30%, Caco‐2 > −0.4, and drug‐likeness (DL) ≥ 0.18 were used as the screening thresholds to screen the oral absorbable and pharmacologically active components in FGWYD [17] (Table 1).

### 2.2. FGWYD Targets and VD Genes

The targets of the FGWYD components were collected from TCMSP [18]. The VD genes were collected from the OMIM database (http://omim.org/) [19], Genecards (http://www.genecards.org) [20], and references [21–25]. The UniProt database (https://www.uniprot.org/) is used to convert target protein names into corresponding official gene names (Table S1 and Table S2).

### 2.3. Network Construction and Analysis Methods

FGWYD potential targets and VD genes were imported into the String database (https://string-db.org/) [26], the species was limited to “*Homo sapiens*,” isolated targets were removed, and the protein-protein interaction (PPI) data was obtained based on the confidence level ≥ 0.4. The analysis results were saved as TSV format files and imported into Cytoscape 3.7.0 software for network construction. The tightly connected part of the PPI network is considered a cluster. In a biological network, cluster may represent a biological module related to disease occurrence or drug treatment. The cluster in the PPI network was detected by MCODE [27]. The target protein gene list was imported into the DAVID database (https://david.ncifcrf.gov/summary.jsp) [28], the species was limited to “*Homo sapiens*,” and Gene Ontology (GO) enrichment analysis and KEGG (Kyoto Encyclopedia of Genes and Genomes) signaling pathway analysis were performed.

### 2.4. Experimental Materials

#### 2.4.1. Experimental Animal

Sixty (60) male SD rats that were specific pathogen-free (SPF) grade were purchased and placed in the Experimental Animal Center of Hunan University of Chinese Medicine (Qualification Certificate No.: HNASLKJ20113616). Animal experiments were approved by the Animal Ethics Committee of Hunan University of Chinese Medicine and were in accordance with the National Institute of Health's Guide for the Care and Use of Laboratory Animals.

#### 2.4.2. Experimental Drugs

FGWYD is composed of *Aconiti Lateralis Radix Praeparata* 15 g, *Zingiberis Rhizoma* 15 g, *Acoritataninowii Rhizoma* 15 g, *Epimrdii Herba* 15 g, *Cinnamomi Ramulus* 15 g, *Morindae Officinalis Radix* 15 g, *Arum Ternatum Thunb.* 15 g, *Panax Ginseng C. A. Mey.* 15 g, *Panax Notoginseng* (*Burk.*) *F. H. Chen Ex C. Chow* 15 g, and *Radix Rhei Et Rhizome* 6 g. The medicinal materials were purchased from the Pharmaceutical Factory of the First Affiliated Hospital of Hunan University of Chinese Medicine. The medicinal materials of FGWYD were extracted twice; the first time was extracted with 10 times the volume of water for 2 hours, and the second time was extracted with 8 times the volume of water for 1.5 hours. The medicinal solution is filtered, combined, and concentrated to contain crude drug 1 g/mL, stored at 4°C. During the experiment, FGWYD was diluted with double-distilled water to an appropriate concentration. Piracetam tablets (Naofukang, NFK) were purchased from Shanghai Xinyi Pharmaceutical Co., Ltd. (Guo Yao Zhunzi H31020714). The reference substance benzoylaconitine (batch number: CHB190207), benzoylmesaconine (batch number: CHB200120), and benzoylhypacoitine (batch number: CHB200201) were purchased from Chengdu Croma Biotechnology Co., Ltd. The quality scores of all controls were ≥98%.

#### 2.4.3. Reagents and Instruments

Instruments: electrothermal constant temperature incubator (Shanghai Yuejin Medical Devices Co., Ltd., HH.B11.360), electrothermal constant drying oven (Shanghai Yuejin Medical Devices Co., Ltd., GZX-DH.400), UVP gel imaging system (Thermo Fisher Scientific, CA91786 USA UVP GDS-8000 System), electrophoresis system (Beijing Liuyi Biological Technology Co., Ltd., DYCZ-24DN), semidry film transfer system (ATTO, WSE-4040), transfer decolorization shaker (Haimen Qilin Bell Instrument Manufacturing Co., Ltd., TS-8), fluorescence quantitative PCR instrument (ABI, 7500), vortex oscillator (Haimen Qilin Bell Instrument Company, QL-902), and horizontal electrophoresis instrument (Beijing Liuyi Biotechnology Co., Ltd.).

Reagents: RIPA lysate (Beijing Soleil, 80010), SuperReal PreMix Plus (SYBR Green) (Tiangen Biotechnology Co., Ltd.), and DL2000 DNA Marker (TAKARA, 3427A). The malondialdehyde (MDA) determination kit, the Total Antioxidant Capacity (T-AOC) Test Kit, the Lipid Peroxide (LPO) Test Kit, the Glutathione Peroxidase (GSH-Px) Test Kit, and the superoxide dismutase (SOD) determination kit were purchased from Nanjing Jiancheng Bioengineering Company. High-fat feed formula was composed of the following: 3% cholesterol, 0.5% sodium cholate, 0.2% propylthiouracil, 5% sugar, 10% lard, and 81.3% basic feed.

### 2.5. Experimental Methods

#### 2.5.1. FGWYD Quality Control

1. Preparation of FGWYD solution: FGWYD 25 mL is precisely pipetted into a round-bottom flask and evaporated to dryness. The residue of FGWYD was accurately combined with 20 mL chloroform, 4 mL ammonia, and 4 mL methanol; heated to reflux for 2 h; cooled and filtered; then washed with chloroform 3 times, combined with the FGWYD filtrate, and evaporated to dryness in a 60°C water bath
2. Preparation of reference solution: benzoylmesaconine, benzoylaconitine, and benzoylhypacoitine 2.81, 2.33, and 2.75 mg were accurately weighed, respectively, and 0.05% hydrochloric acid-methanol was added to prepare 0.562, 0.466, and 0.550 g/L benzoylmesaconine, benzoylaconitine, and benzoylhypacoitine reference solutions
3. HPLC condition: Waters XTerra MS C18 column (150 mm × 4.6 mm, 5 *μ*m), XTerra MS C18 guard column (20 mm × 4.6 mm, 5 *μ*m). Mobile phase: acetonitrile (a) -0.1 mol·L^−1^ ammonium acetate solution (pH = 6.97) (b); gradient elution; flow rate: 1 mL·min^−1^. Detection wavelength: 235 nm. Injection volume: 10 *μ*L; detection temperature: room temperature

The HPLC results showed that the content of the components in FGWYD was benzoylmesaconine 0.14928 mg/g, benzoylaconitine 0.014736 mg/g, and benzoylhypacoitine 0.021648 mg/g (Figure S1).

#### 2.5.2. Animal Modeling

The rats were fed adaptively for 5 days. On the sixth day, a water maze test was performed to exclude rats with swimming disorders and rats that failed to find a platform. A total of 3 rats were removed. Then, the rats were randomly grouped: 10 rats in the sham operation group, and the remaining 50 rats entered the modeling group. The experimental group was fed a high-fat diet, and at the same time, a one-time intraperitoneal injection of vitamin D3 700,000 IU/kg at the beginning of feeding. The sham operation group was fed basic feed and given the same volume of saline. After the success of the atherosclerosis (AS) model, on the basis of the AS model, the rat VD model was prepared by the bilateral common carotid artery clipping and reperfusion method. In rats from the sham operation group, only neck skin incision was performed without ischemic surgery. The cages were marked with numbers as groups, and intervention drugs were also marked with numbers. The meaning of the number will be kept by a third person before the end of the experiment. Animal experiment operators, data collectors, and statistical analysts were not aware of grouping and intervention drugs.

#### 2.5.3. Animal Grouping and Intervention

One week after modeling, the rats were given intragastric administration after the surgical incision of the rats was completely healed. Fifty (50) successful rats were randomly divided into 5 groups: the FGWYD low-dose group (FGWYD-L, 10 rats), the FGWYD medium-dose group (FGWYD-M, 10 rats), the FGWYD high-dose group (FGWYD-H, 10 rats), the positive control group (NFK, 10 rats), and the model group (10 rats).

The intervention began 4 weeks after modeling, and the dosage was calculated based on the ratio of human and rat body surface area coefficient. The FGWYD low-dose group was given 1.25 g of crude drug/kg, the FGWYD medium-dose group was given 2.5 g of crude drug/kg, and the FGWYD high-dose group was given 5 g of crude drug/kg. The positive drug control group was given piracetam 0.15 g/kg. The sham operation group and the model group were given intragastric administration with the corresponding volume of double-distilled water. The administration lasted 14 days. Two rats died in the model group, FGWYD high-dose group, and positive control group, respectively. Morris water maze experiment was used to test learning and memory ability. HE staining was used to observe the pathological changes of brain tissue.

#### 2.5.4. Morris Water Maze Behavior Test

The pool was equally divided into four quadrants and marked, and four quadrant entry points are marked on the inner wall of the pool, and a platform is placed at the center of the third quadrant of the pool. The pool was filled with water up to about 2 cm above the platform. The ink is added to the water and mixed well to hide the platform. The water in the pool is heated and maintained at around 22°C. A small fixed camera is installed above the pool to track and record the swimming trajectory of rats. The content of Morris water maze detection includes a positioning navigation experiment and a space exploration experiment.

#### 2.5.5. Detection of Nrf2 and HO-1 Protein Expression by Western Blot

After the water maze experiment on rats was completed, fresh brain was collected under low temperature under anesthesia with 1% sodium pentobarbital 35 mg/kg. After extracting the total protein and measuring the concentration, SDS-PAGE electrophoresis was performed. After the transfer, the color was developed with ECL color-developing solution.

#### 2.5.6. NRF2 and ARE Binding Force Test by Electrophoretic Mobility Shift Assay (EMSA)

EMSA was performed after extracting the nucleoprotein. First, the oligonucleotide probe is denatured and annealed into a double strand (94°C for 5 min, gradually returning to room temperature), and the annealing effect is checked by 12% PAGE gel electrophoresis, diluted and aliquoted, and stored at -20°C for use. The EMSA operation was carried out according to the operating procedures of the LightShift™ Chemiluminescent EMSA Kit (Pierce Inc.). The oligonucleotide sequences used in the experiment are shown in Table 2.

#### 2.5.7. Detection of HO-1 mRNA Expression by Real-Time PCR

Total RNA in tissue was extracted with TRIzol and was reversed transcribed into cDNA. The real-time PCR reaction system is composed of the following: 2x SuperReal PreMix Plus 10 *μ*L, upstream primer (10 *μ*M) 0.6 *μ*L, downstream primer (10 *μ*M) 0.6 *μ*L, cDNA 100 mg, 50x ROX Reference Dye 0.4 *μ*L, and RNase-Free ddH_2_O to 20 *μ*L. Program setting is as follows: predenaturation at 95°C for 15 min once, at 95°C for 10 s, at 58°C for 20 s, and at 72°C for 30 s, for 40 cycles. The primer is shown in Table 3.

#### 2.5.8. Detection of the MDA, SOD, GSH-Px, and LPO Contents and Total Antioxidant Capacity (T-AOC) of Hippocampus

The MDA, SOD, GSH-Px, and LPO contents and T-AOC of the hippocampus were determined strictly in accordance with the kit instructions.

### 2.6. Proteomics Methods

#### 2.6.1. Total Protein Extraction

Under the anesthesia with 1% sodium pentobarbital 35 mg/kg, the hippocampus of the rats was taken out and frozen in the refrigerator at -80°C. During the experiment, the rat hippocampus sample was taken out from the refrigerator at -80°C, put into a liquid nitrogen precooled mortar, and added liquid nitrogen to grind to a powder. Four times the volume of lysis buffer was added to the sample and placed in an ultrasonic disruptor for ultrasonic lysis. The above sample was centrifuged at 12000 g for 10 min at 4°C, and the supernatant was extracted, and then the protein concentration was determined using the Bradford kit.

#### 2.6.2. Isobaric Tags for Relative and Absolute Quantification

After the protein was digested by trypsin, the peptide was desalted with StrataXC18 (Phenomenex) and then freeze-dried in vacuo. The peptide was dissolved with 0.5 M TEAB, and then they were labeled according to the instructions of the TMT kit. The sample marking information is shown in Table 4.

#### 2.6.3. HPLC Classification

The peptides were fractionated by high pH reverse HPLC, and the column was Agilent 300Extend C18 (5 *μ*m particle size, 4.6 mm inner diameter, 250 mm long).

#### 2.6.4. Liquid Chromatography-Mass Spectrometry Analysis

The peptides were dissolved in liquid chromatography mobile phase A and separated using the EASY-nLC 1000 ultra-high-performance liquid system. After separation by ultra-high-performance liquid phase system, the peptides were injected into the NSI ion source for ionization and then analyzed by Q Exactive mass spectrometry. The secondary mass spectrometry data was retrieved using Sequest software integrated with Proteome Discoverer (version 1.3, Thermo Fisher Scientific). Retrieval parameter settings are as follows: the database is the rat proteome database in Uniprot, named 10116-PrRattusNorvegicus-0171215-9795 (Proteome ID: UP000002494) (29795 sequences).

#### 2.6.5. Mass Spectrometry Quality Control Detection

**Figure 2:**
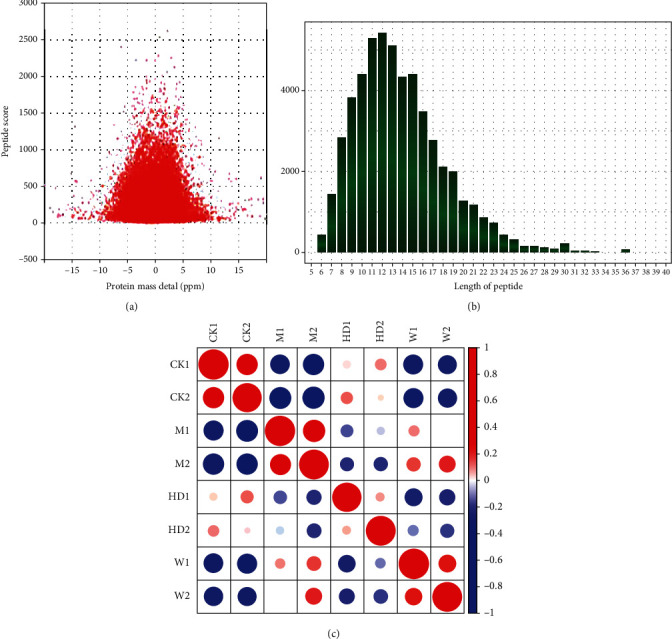
Mass spectrometry quality control ((a) quality bias distribution of peptide segment; (b) identified peptide distribution; (c) sample repeatability test; CK1: control group 1; CK2: control group 2; M1: model group 1; M2: model group 2; HD1: FGWYD group 1; HD2: FGWYD group 2; W1: positive control group 1; W2: positive control group 2).

The quality control results of mass spectrometry data are shown in Figure 2. First, we detected all the identified mass errors that were too short (mass error) (Figure 2(a)). The mass error is centered on 0 and concentrated in the range below 10 ppm, indicating that the mass error meets the requirements. Secondly, most of the peptides are distributed between 8 and 20 amino acid residues (Figure 2(b)), which is in accordance with the rule of trypsin digestion of peptides, indicating that the sample preparation meets the standard. The color in Figure 2(c) represents the correlation coefficient of protein quantification between samples. The higher the correlation coefficient between samples in the same group, the better the repeatability and the redder the color. CK1 (control group 1) and CK2 (control group 2), M1 (model group 1) and M2 (model group 2), HD1 (FGWYD group 1) and HD2 (FGWYD group 2), and W1 (positive control group 1) and W2 (positive control group 2) have higher repeatability among samples.

### 2.7. Statistical Analysis

All data were analyzed using SPSS17.0. Quantitative data is expressed as mean and standard deviation (*x* ± *s*). If the data conforms to the normal distribution, *t*-test analysis is used for comparison between two groups, and analysis of variance is used for comparison between multiple groups. A nonparametric test is used for data that does not conform to the normal distribution. The *χ*^2^ test is used for counting data, and the rank sum test is used for ranking data. *P* < 0.05 was considered statistically significant.

## 3. Results and Discussion

### 3.1. FGWYD Targets and VD Genes

**Figure 3:**
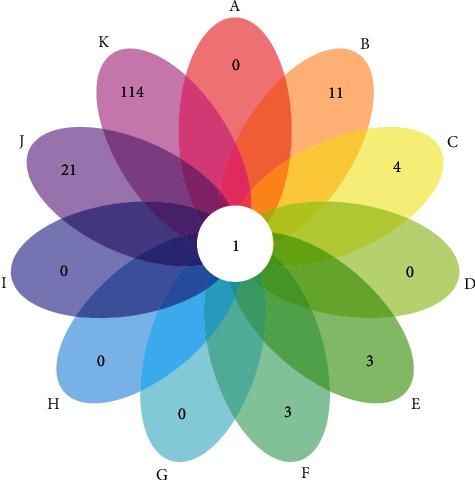
Venn diagram of FGWYD targets and VD genes ((A) *Morindae Officinalis Radix*; (B) *Arum Ternatum Thunb.*; (C) *Radix Rhei Et Rhizome*; (D) *Aconiti Lateralis Radix Praeparata*; (E) *Cinnamomi Ramulus*; (F) *Panax Ginseng C. A. Mey.*; (G) *Panax Notoginseng* (*Burk.*) *F. H. Chen Ex C. Chow*; (H) *Zingiber Officinale Roscoe*; (I) *Acoritataninowii Rhizoma*; (J) *Epimrdii Herba*; (K) VD).

A total of 73 FGWYD components with 245 FGWYD targets were obtained from TCMSP. The VD-related genes with relevance score > 5.5 were selected for sequence research. A total of 145 VD genes were obtained from that database. Among those FGWYD targets, *Morindae Officinalis Radix* gets 34 potential targets, *Arum Ternatum Thunb.* gets 84 targets, *Radix Rhei Et Rhizome* gets 44 targets, *Aconiti Lateralis Radix Praeparata* gets 21 targets, *Cinnamomi Ramulus* gets 20 targets, *Panax Ginseng C. A. Mey.* gets 99 targets, *Panax Notoginseng* (*Burk.*) *F. H. Chen Ex C. Chow* gets 168 targets, *Zingiber Officinale Roscoe* gets 35 targets, *Acoritataninowii Rhizoma* gets 80 targets, and *Epimrdii Herba* gets 218 targets. There is overlap between the target set of FGWYD herbs and the VD gene set (Figure 3).

**Figure 4:**
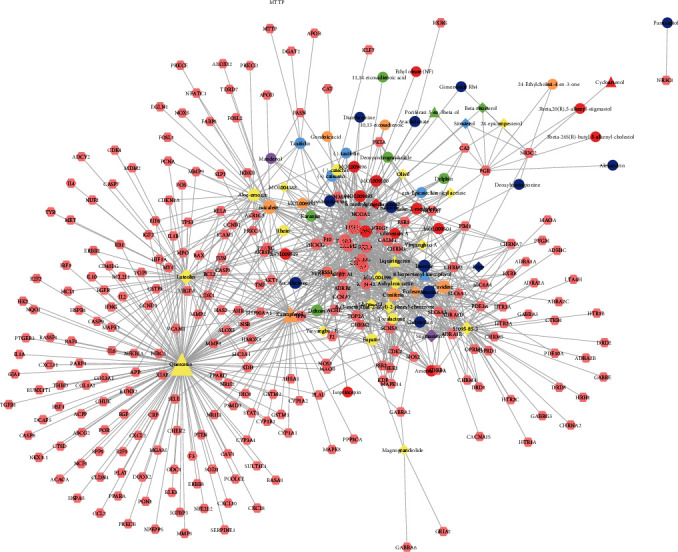
Component-target network of FGWYD. (Pink hexagons stand for FGWYD targets. Red, orange, yellow, green, blue, indigo, and purple circles stand for components of *Morindae Officinalis Radix*, *Arum Ternatum Thunb.*, *Radix Rhei Et Rhizome*, *Aconiti Lateralis Radix Praeparata*, *Cinnamomi Ramulus*, *Panax Ginseng C. A. Mey.*, and *Panax Notoginseng* (*Burk.*) *F. H. Chen Ex C. Chow*, respectively. Red, orange, and yellow diamonds stand for compounds of *Zingiber Officinale Roscoe*, *Acoritataninowii Rhizoma*, and *Epimrdii Herba*, respectively. Yellow diamond stands for common component of *Morindae Officinalis Radix*, *Arum Ternatum Thunb.*, *Radix Rhei Et Rhizome*, *Aconiti Lateralis Radix Praeparata*, *Cinnamomi Ramulus*, *Panax Ginseng C. A. Mey.*, *Panax Notoginseng* (*Burk.*) *F. H. Chen Ex C. Chow*, and *Zingiber Officinale Roscoe*. Green diamond stands for common component of *Morindae Officinalis Radix*, *Aconiti Lateralis Radix Praeparata*, *Cinnamomi Ramulus*, and *Epimrdii Herba*. Blue diamond stands for common component of *Morindae Officinalis Radix*, *Panax Ginseng C. A. Mey.*, and *Panax Notoginseng* (*Burk.*) *F. H. Chen Ex C. Chow*. Indigo diamond stands for common component of *Morindae Officinalis Radix*, *Panax Ginseng C. A. Mey.*, and *Panax Notoginseng* (*Burk.*) *F. H. Chen Ex C. Chow*. Purple diamond stands for common component of *Arum Ternatum Thunb.*, *Panax Ginseng C. A. Mey.*, *Panax Notoginseng* (*Burk.*) *F. H. Chen Ex C. Chow*, and *Zingiber Officinale Roscoe*. Red triangle stands for common component of *Arum Ternatum Thunb.* and *Acoritataninowii Rhizoma*. Orange triangle stands for common component of *Panax Ginseng C. A. Mey.*, *Acoritataninowii Rhizoma*, and *Epimrdii Herba*. Yellow triangle stands for common component of *Panax Notoginseng* (*Burk.*) *F. H. Chen Ex C. Chow* and *Epimrdii Herba*. Green triangle stands for common component of *Zingiber Officinale Roscoe* and *Epimrdii Herba*. Blue triangle stands for common component of *Acoritataninowii Rhizoma* and *Epimrdii Herba*.)

The FGWYD components and FGWYD targets were input into Cytoscape to construct component-target network of FGWYD. This network consists of 73 FGWYD component nodes, 245 target nodes and 964 edges (Figure 4). The nodes with higher degree was bigger in this network.

### 3.2. FGWYD-VD PPI Network Analysis

#### 3.2.1. FGWYD-VD PPI Network Construct

**Figure 5:**
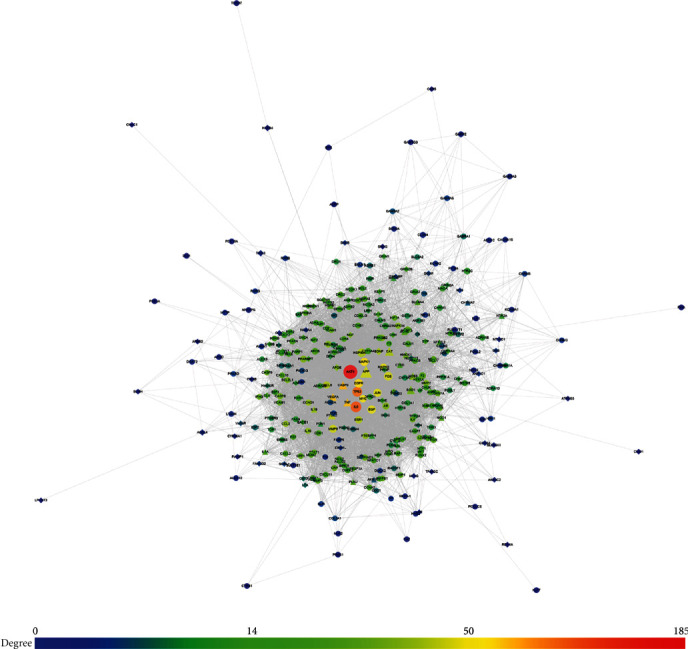
FGWYD-VD PPI network (circles stand for FGWYD targets; diamonds stand for VD genes; triangles stand for FGWYD-VD targets; the color of the nodes was related to the degree; the size of the nodes was positively related to their betweenness).

**Figure 6:**
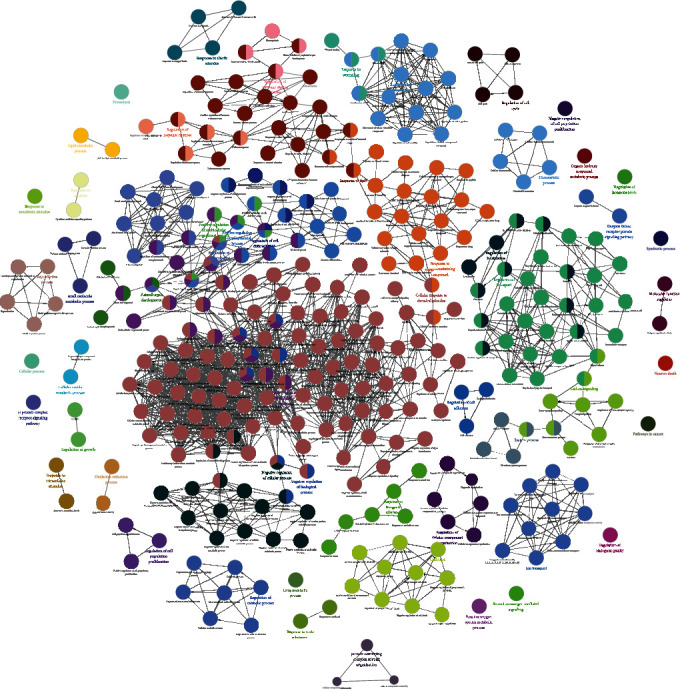
The primary enrichment analysis results visualized by ClueGO.

The FGWYD targets, VD genes, and their PPI data were input into Cytoscape to construct the FGWYD-VD PPI network. This network is composed of 512 nodes (213 compound targets, 267 VP genes, and 32 BHD-VP targets) and 8468 edges (Figure 3). The targets are arranged in descending order of degree. The top 10 targets in each target set are: (1) FGWYD target set: AKT1 (185 edges), IL6 (163 edges), TP53 (158 edges), EGFR (135 edges), MAPK1 (134 edges), MYC (130 edges), EGF (126 edges), MAPK8 (125 edges), JUN (125 edges), and FOS (120 edges); (2) VD target set: BDNF (104 edges), APOE (89 edges), NGF (88 edges), ACE (71 edges), SP1 (65 edges), TNFRSF1A (61 edges), GFAP (51 edges), CD40 (49 edges), MAPT (48 edges), and CLU (47 edges); and (3) FGWYD-VD target set: CASP3 (143 edges), TNF (141 edges), VEGFA (140 edges), APP (119 edges), PTGS2 (114 edges), CAT (107 edges), IL1B (99 edges), NOS3 (90 edges), HMOX1 (84 edges), and SOD1 (82 edges) (Figure 5). The primary enrichment analysis results are shown in Figure 6.

#### 3.2.2. Biological Processes of FGWYD-VD PPI Network Construct

**Figure 7:**
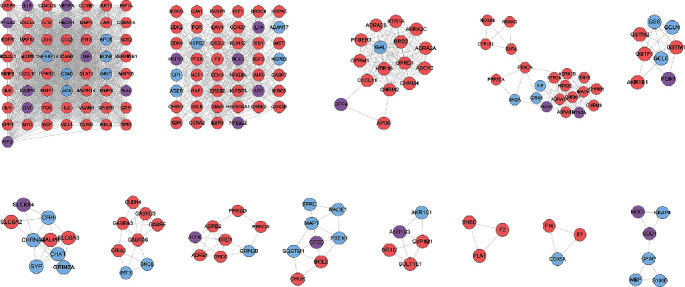
Clusters of FGWYD-VD PPI network (pink, blue, and purple circles stand for FGWYD targets, VD targets, and FGWYD-VD targets, respectively).

The FGWYD-VD PPI network was analyzed by MCODE and thirteen clusters were obtained (Table 5 and Figure 7). The targets in the clusters were input into DAVID to perform GO enrichment analysis, and got a lot of biological processes.

**Figure 8:**
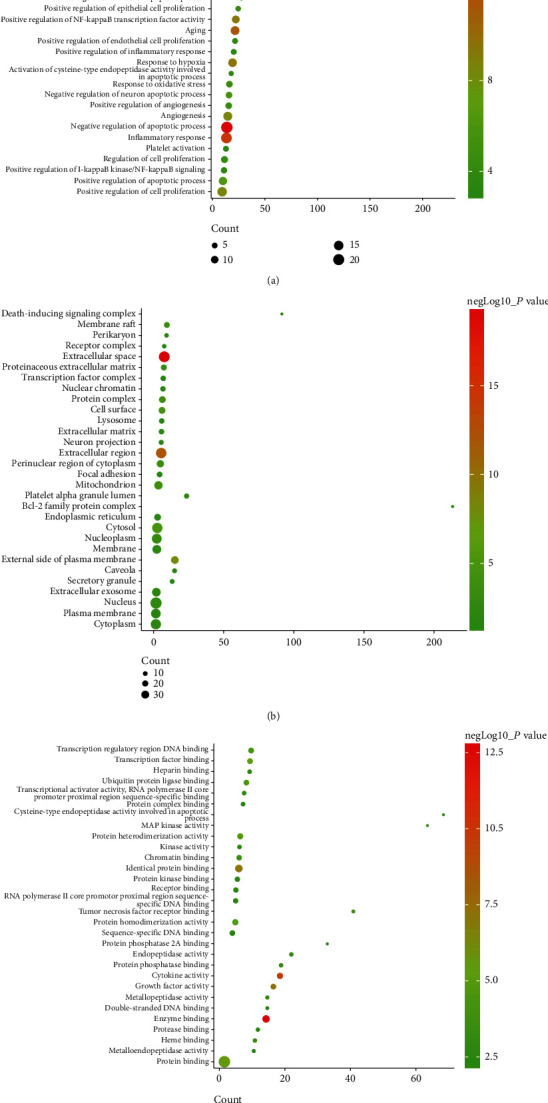
Bubble chart of cluster 1 ((a) biological processes; (b) cell components; (c) molecular function; *x*-axis stands for fold enrichment).

Cluster 1 is mainly related to apoptosis, inflammation, hypoxic response, angiogenesis, neuronal apoptosis, and oxidative stress. Cluster 2 is mainly related to angiogenesis, endoplasmic reticulum stress, inflammation, angiogenesis, and redox. Cluster 3 is mainly related to smooth muscle contraction, chemical synapses, synaptic transmission, and platelet activation. Cluster 4 is mainly related to synaptic transmission, neurotransmitter synthesis and catabolism, vasoconstriction, neuronal synaptic plasticity, and redox. Cluster 5 is mainly related to glutathione anabolism, oxidative stress, and mitochondrial depolarization. Cluster 6 is mainly related to neurotransmitter metabolism and synthesis. Cluster 7 is mainly related to chemical synaptic transmission, dopamine uptake and synaptic transmission, and hypoxia. Cluster 8 is mainly related to synaptic transmission and nerve impulse. Cluster 9 is mainly related to endoplasmic reticulum calcium homeostasis, negative regulation of neuronal apoptosis, and autophagy. Cluster 10 is mainly related to steroid metabolism. Cluster 11 is mainly related to coagulation, fibrinolysis, and platelet activation. Cluster 12 is mainly related to blood coagulation. Cluster 13 is mainly related to oxygen free radicals, synapse enhancement, and apoptosis (Table S3). The biological processes, cell components, and molecular function of cluster 1 is shown in Figure 8 as an example.

#### 3.2.3. Signaling Pathway of FGWYD-VD PPI Network

**Figure 9:**
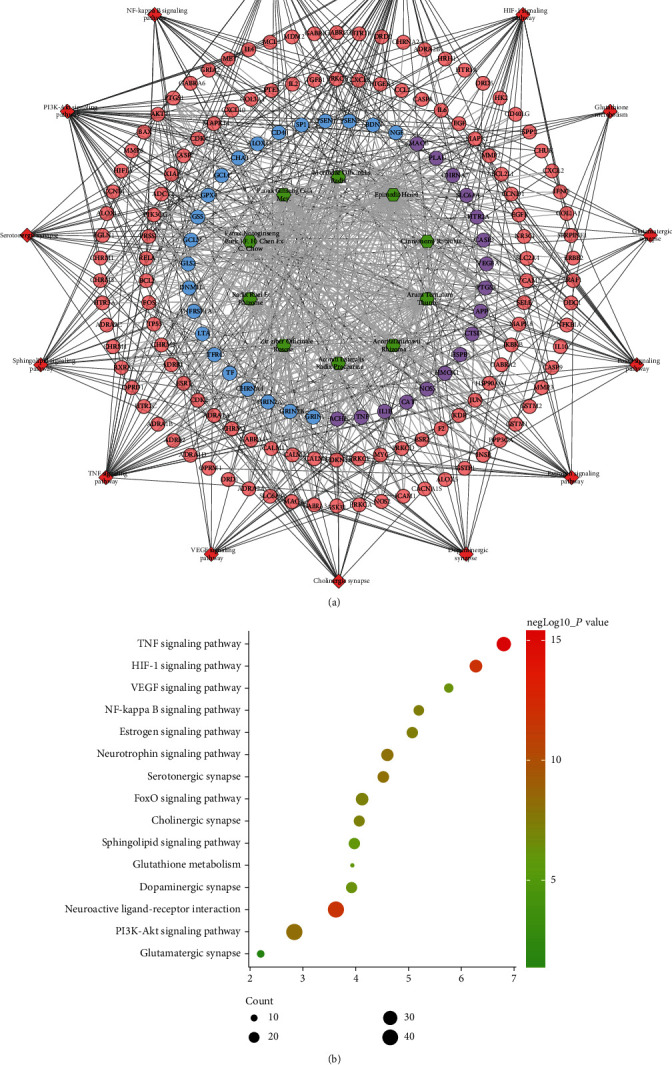
Signaling pathway of FGWYD-VD PPI network ((a) herb-target-signaling pathway network; blue, pink, and purple circles stand for the FGWYD target, VD genes, and the FGWYD-VD target, respectively; red diamonds stand for pathway; green hexagons stand for herb; (b) bubble chart of signaling pathway; *x*-axis stands for fold enrichment).

The targets and genes in the FGWYD-VD PPI network was input into DAVID to perform pathway enrichment analysis, and it returned fifteen core VD-related pathways (Figure 9(a)). The top 10 signaling pathways are as follows: the TNF signaling pathway, the HIF-1 signaling pathway, neuroactive ligand-receptor interaction, the PI3K-Akt signaling pathway, the neurotrophin signaling pathway, the estrogen signaling pathway, the NF-kappa B signaling pathway, the FoxO signaling pathway, the serotonergic synapse, and the VEGF signaling pathway (Figure 9(b)). The details of the signaling pathway are shown in Table S4.

**Figure 10:**
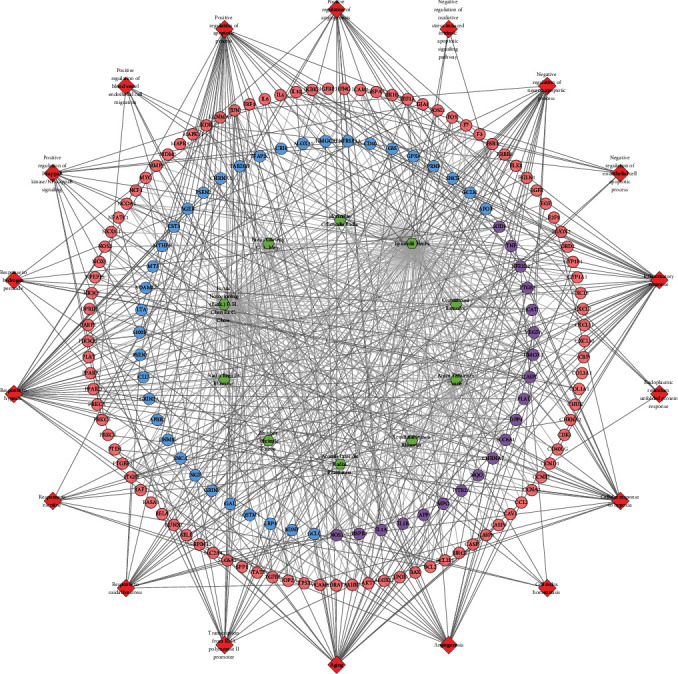
Herb-target-biological process network (blue, pink, and purple circles stand for the FGWYD target, VD genes, and the FGWYD-VD target, respectively; red diamonds stand for the pathway; green hexagons stand for herb.)

Current research shows that VD is currently the only preventable senile dementia, which is characterized by histopathological damage and progressive mental decline caused by hypoxic or hemorrhagic brain injury [29]. The hippocampus is an important structure for learning and memory, and it is extremely sensitive to cerebral ischemia and hypoxia. Cerebral ischemia and hypoxia can easily cause hippocampal neuron apoptosis and decrease learning and memory ability [30]. The currently generally accepted pathogenesis of VD includes cholinergic system dysfunction (acetylcholine deficiency or decreased choline acetyltransferase activity), neurosynaptic changes (decreased synaptic plasticity), excitatory amino acid toxicity damage, oxidative stress injury, and neuronal apoptosis [31–33]. New research shows that cerebrovascular changes may be involved in neurological dysfunction and cognitive impairment. Vascular endothelial dysfunction and neurovascular unit decoupling mediated by ischemia, hypoxia, oxidative stress, inflammation, and other factors can lead to neuronal damage or apoptosis, and ultimately cause cognitive impairment and neurodegenerative changes [34, 35]. In summary, the etiology and pathogenesis of VD are complex, and searching and determining the key signal pathways or targets for the occurrence and development of VD are particularly important for the development of specific drugs. The application of systems biology and network pharmacology technology also has important hints for follow-up experimental research [36]. Based on the acquisition of FGWYD targets and VD-related genes, this study used bioinformatics techniques to analyze a total of 13 clusters and 14 signal pathways that may be involved in the prevention and treatment of VD by FGWYD and we found that Nrf2 (NFE2L2) and HO-1 (HMOX1) may play an important role in the treatment of VD by FGWYD (Figure 10). The integrated analysis of network biology modularity shows that the VD-related pathological biological modules mainly regulated by FGWYD are as follows: inflammation module, oxidative stress, synaptic plasticity regulation module, neuronal apoptosis module, and angiogenesis module.

### 3.3. The Results of Morris Water Maze Behavior Test

#### 3.3.1. Latent Period Results of Positioning Navigation Experiment

**Figure 11:**
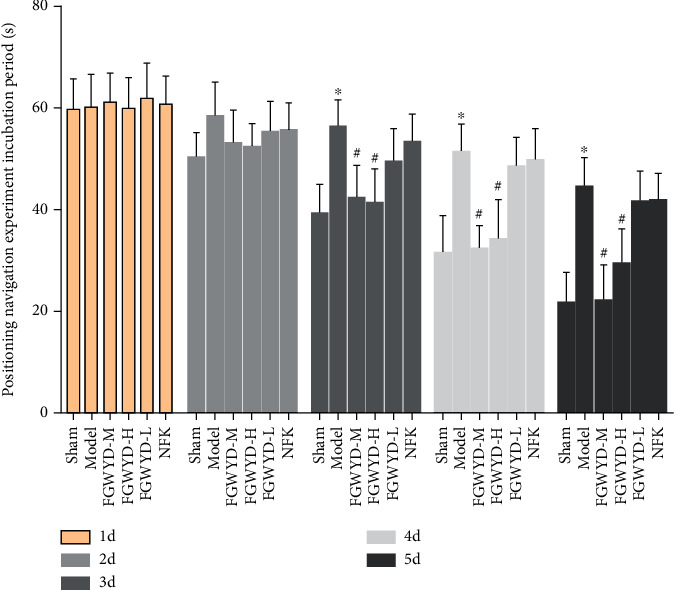
Latent period results of positioning navigation experiment (compared with sham operation group, ^∗^*P* < 0.01; compared with the model group, ^#^*P* < 0.01).

In the positioning navigation experiment, there was no significant difference in the incubation period of the rats in each group in the first two days, and there was no statistical difference. From the third day, compared with the model group, the latent period of the FGWYD medium-dose group, the high-dose group, and the sham operation group was significantly shortened (*P* < 0.01) (Figure 11).

#### 3.3.2. Space Probe Experiment Results

**Figure 12:**
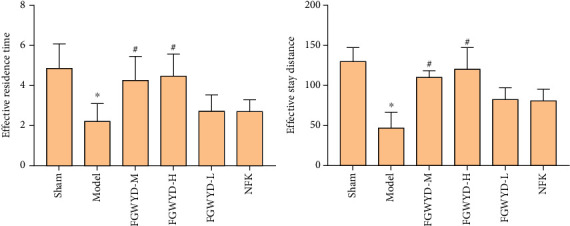
Space probe experiment results (compared with the sham operation group, ^∗^*P* < 0.01; compared with the model group, ^#^*P* < 0.01).

In the space probe experiment, the effective residence time of the FGWYD medium- and high-dose groups was significantly prolonged, and there was no statistical difference compared with the sham operation group (*P* > 0.05). Compared with the model group, the effective residence time of other groups was significantly different (*P* < 0.01) (Figure 12).

### 3.4. Pathological Changes

#### 3.4.1. Sham Operation Group

**Figure 13:**
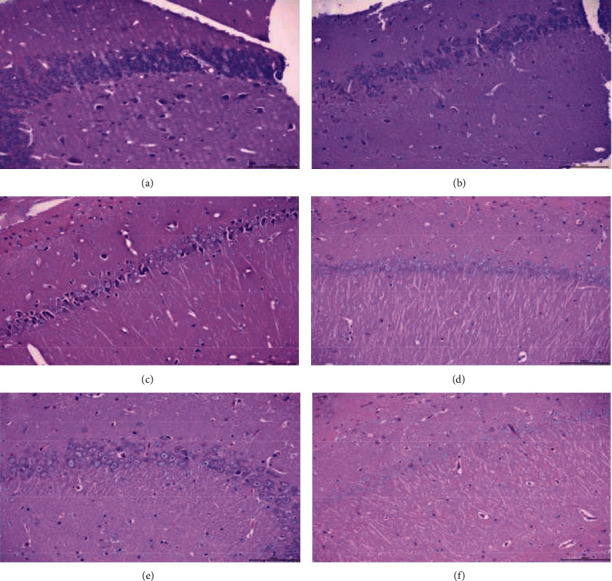
Pathological changes (HE staining, 200x; (a) sham operation group; (b) model group; (c) model group; (d) FGWYD high-dose group; (e) FGWYD low-dose group; (f) positive control group).

In the sham operation group, the structure of the hippocampus is normal; the neurons in the hippocampus are tightly arranged, the cell structure is clear, the nucleus has no obvious pyknosis, the surrounding stroma has no obvious edema, the blood vessels have no obvious expansion, and the tissue has no obvious inflammatory cell infiltration (Figure 13(a)).

#### 3.4.2. Model Group

In the model group, the structure of the hippocampus is abnormal. The number of neurons in the hippocampus is reduced, some neurons are arranged in disorder, and the nuclei are constricted. Some neurons were pyknotic and deeply stained, there was no obvious edema in the surrounding interstitium, no obvious expansion of blood vessels, and no obvious inflammatory cell infiltration in the tissue (Figures 13(b) and 13(c)).

#### 3.4.3. FGWYD High-Dose Group

In the FGWYD high-dose group, the structure of the hippocampus is normal. The cells are arranged neatly, there is no obvious pyknosis and deep staining, no obvious edema in the surrounding interstitium, no obvious expansion of blood vessels, and no obvious inflammatory cell infiltration in the tissue (Figure 13(d)).

#### 3.4.4. FGWYD Low-Dose Group

In the FGWYD low-dose group, the structure of the hippocampus is abnormal. It can be seen that individual neurons are pyknotic and deeply stained, the cell arrangement is basically neat, there is no obvious disorder, and the number of cells is not significantly reduced (Figure 13(e)).

#### 3.4.5. Positive Control Group

In the positive control group, the structure of the hippocampus is abnormal. It can be seen that individual neurons are pyknotic and deeply stained; the cell arrangement is basically neat, without obvious disorder, and the number of cells is not significantly reduced (Figure 13(f)).

### 3.5. Proteomics Results

#### 3.5.1. Differential Expression Protein Identification

Compared with the sham operation group, 23 proteins were upregulated and 17 proteins were downregulated in the 2-fold difference protein (*P* < 0.05) in the model group (Table 6). Compared with the model group, 16 proteins were upregulated and 10 proteins were downregulated in the FGWYD group with more than 2-fold differential protein (*P* < 0.05) (Table 7).

#### 3.5.2. Cluster Diagram of Differential Protein Expression Levels

**Figure 14:**
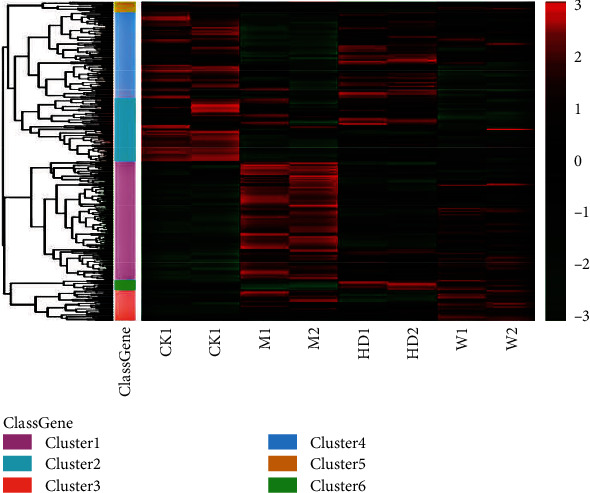
Cluster diagram of differential protein expression levels (CK1: control group 1; CK2: control group 2; M1: model group 1; M2: model group 2; HD1: FGWYD group 1; HD2: FGWYD group 2; W1: positive control group 1; W2: positive control group 2).

In the control group, the expression of related genes in cluster 1 was downregulated, and the expression of related proteins in cluster 2 was upregulated. In the model group, the expression of related proteins in cluster1 was upregulated, and the expression of related proteins in cluster2 was downregulated. In the FGWYD group, the expression of related proteins in cluster 1 was downregulated, and the expression of related proteins in cluster 2 was downregulated. The protein difference between the model group and the control group was obvious. After FGWYD, the protein expression recovery of the FGWYD group was similar to that of the control group. We can think that the modeling of the VD rat model group was successful, and the expression of related abnormal proteins was restored after FGWYD treatment (Figure 14).

#### 3.5.3. Bioinformatics Analysis

**Figure 15:**
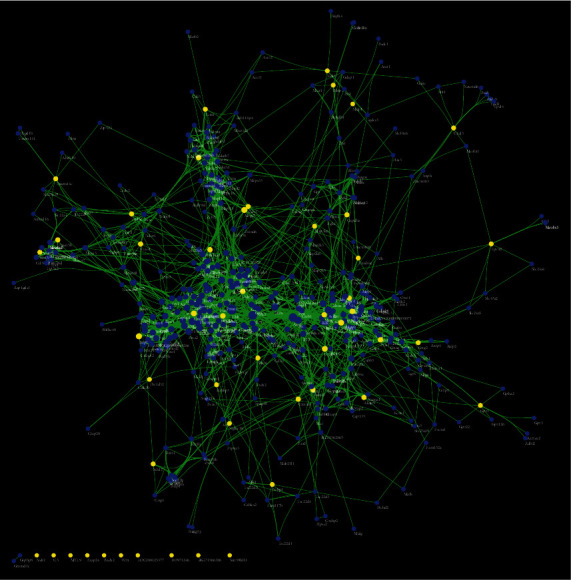
Differential expression protein-other rat protein PPI network (yellow circles stand for differential expression protein circle; blue circles stand for other rat protein circle).

**Figure 16:**
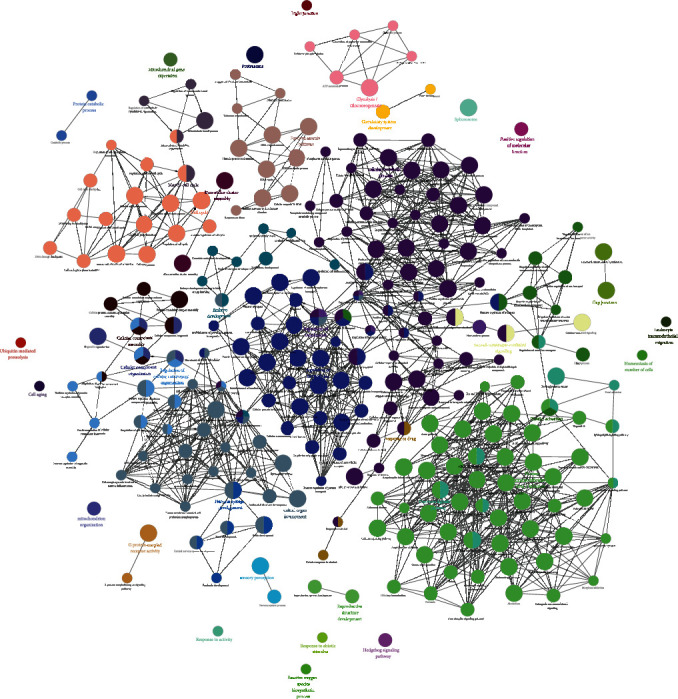
The primary enrichment results of differential expression protein-other rat protein PPI network.

**Figure 17:**
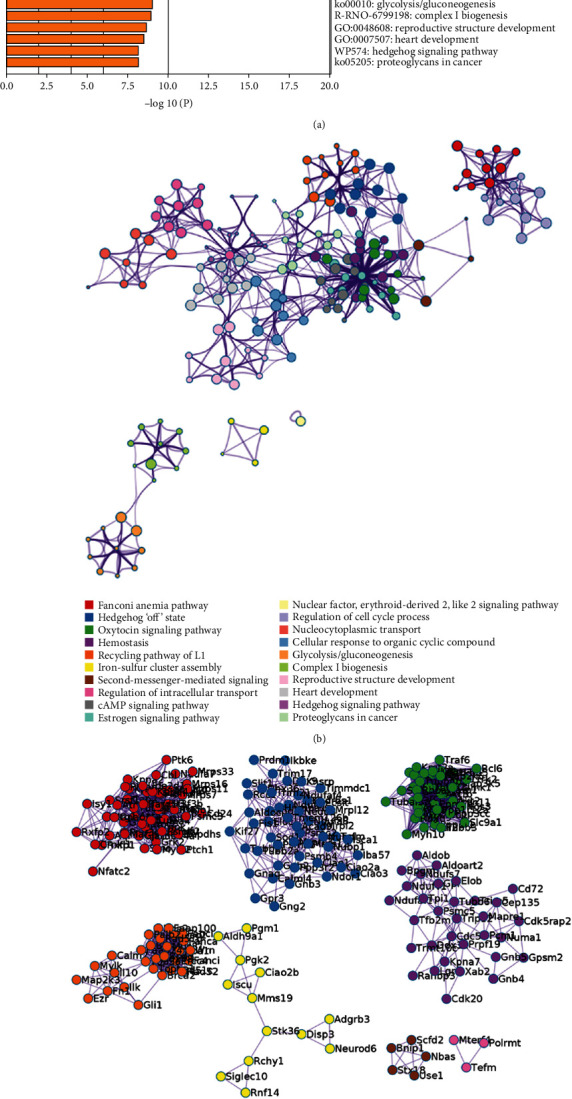
The metascape analysis results ((a) top enrichment results of proteomics proteins' PPI network; (b) PPI network colored by enrichment results; (c) clusters of PPI network).

The differentially expressed proteins of the sham operation/model group and the FGWYD/model group are combined and deduplicated. Then, they were imported into String, and the species was defined as “*Rattus norvegicus*,” and other rat proteins related to these differentially expressed proteins and the PPI data were collected. Cytoscape was utilized to construct and analyze the network (Figure 15). This network consists of 448 nodes and 3137 edges (Table S5). The primary enrichment analysis results are shown in Figure 16. The clusters of this network are shown in Figure 17.

**Figure 18:**
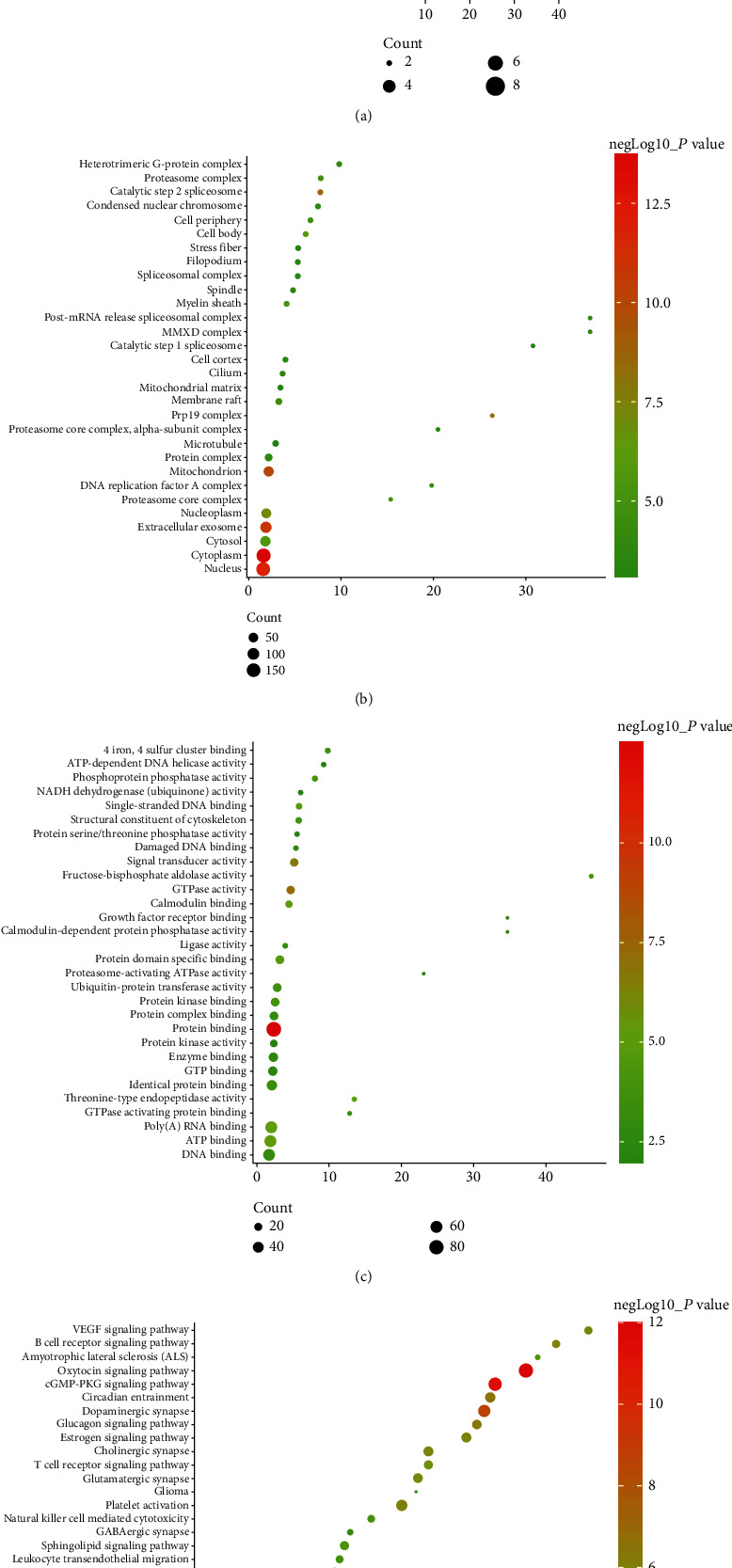
Bubble chart of bioinformatics analysis results ((a) biological processes; (b) cell components; (c) molecular function; (d) signaling pathways; *x*-axis stands for fold enrichment).

The proteins were input into DAVID to perform GO enrichment analysis and pathway enrichment analysis. The results showed that FGWYD may regulate VD-related biological processes and signaling pathways such as iron-sulfur cluster assembly, calcineurin-NFAT signaling cascade, glycolytic process, cellular response to platelet-derived growth factor stimulus, smoothened signaling pathway involved in dorsal/ventral neural tube patterning, oxytocin signaling pathway, cGMP-PKG signaling pathway, dopaminergic synapse, circadian entrainment, glucagon signaling pathway, platelet activation, and B cell receptor signaling pathway (Figure 18 and Table S6).

In order to further illustrate the mechanism of FGWYD intervention in VD in animal models, this study combined isobaric tags for relative and absolute quantification (ITRAQ) with liquid chromatography-mass spectrometry to identify differential proteins and bioinformatics analysis in the hippocampus of VD rats. This provides new ideas for systematic research on the pathogenesis of VD and TCM treatment of VD. The biological function annotations of differentially expressed proteins show that FGWYD regulates the main biological processes of VD: iron metabolism (GO: 0016226), oxidative respiratory chain and other forms of mitochondrial energy metabolism (GO: 0032981 and GO: 0032543), and neuronal apoptosis (GO: 0070997). The signaling pathways of FGWYD for regulating VD mainly involve the following: nerve synapse remodeling and neurotransmitter synthesis and transmission, oxidative stress, calcium regulation signaling pathway, Alzheimer's disease, and neurotrophin signaling pathway).

### 3.6. Effect of FGWYD on the Expression of Nrf2 Protein in VD Rats

#### 3.6.1. Nrf2 Protein Expression in the Nucleus

**Figure 19:**
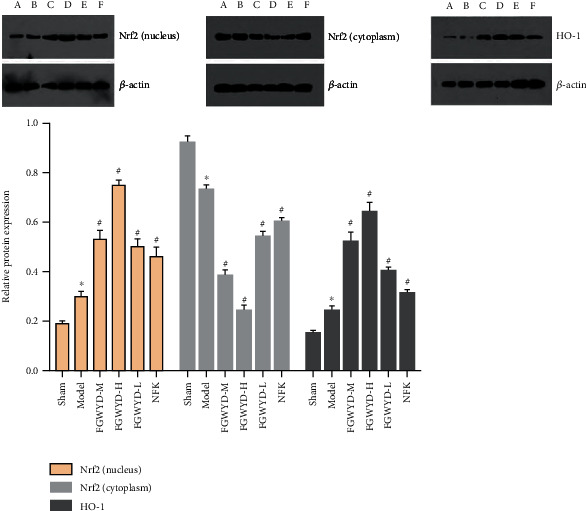
Effect of FGWYD on the expression of Nrf2 and HO-1 protein in VD rats ((A) sham operation group; (B) model group; (C) FGWYD medium-dose group; (D) FGWYD high-dose group; (E) FGWYD low-dose group; (F) positive control group; compared with sham operation group, ^∗^*P* < 0.01; compared with model group, ^#^*P* < 0.01).

The Nrf2 protein content in the hippocampal nuclei of each group was statistically significantly different (*P* < 0.01); there was a statistical difference between the FGWYD low-dose group and the positive group (*P* < 0.05). After AS cerebral ischemia-reperfusion injury, the Nrf2 protein pathway in the rat hippocampal nucleus is activated, and the Nrf2 protein enters the nucleus from the cytoplasm, and its expression increases in the nucleus. The expression of the Nrf2 protein in the hippocampal nucleus of rats in the model group increased. After medication, the Nrf2 protein in the hippocampus of the rat increased, and the FGWYD medium- and high-dose groups were the most significant (Figure 19).

#### 3.6.2. Cytoplasmic Nrf2 Protein Expression

The Nrf2 protein content in the hippocampal nuclei of each group was statistically significantly different (*P* < 0.01). Among them, there was a statistical difference between the FGWYD low-dose group and the positive group (*P* < 0.05). Experiments have shown that after AS cerebral ischemia-reperfusion injury, the Nrf2 protein pathway in the rat hippocampal nucleus is activated, and the Nrf2 protein enters the nucleus from the cytoplasm, and its expression in the cytoplasm decreases. The expression of the Nrf2 protein in the hippocampus cytoplasm of rats in the model group was reduced. After medication, most of the Nrf2 protein in the hippocampus of rats entered the nucleus from the cytoplasm, and the FGWYD medium- and high-dose groups were the most significant (Figure 19).

### 3.7. Effect of FGWYD on the Expression of HO-1 Protein in VD Rats

Compared with the sham operation group, the HO-1 protein content in the hippocampus of each group was statistically significantly different (*P* < 0.01). Experiments have shown that after AS cerebral ischemia-reperfusion injury, the Nrf2 protein pathway in the rat hippocampus nucleus is activated and the downstream protein HO-1 is activated at the same time to increase its expression, and the FGWYD medium- and high-dose groups were the most significant (Figure 19).

### 3.8. The Results of EMSA

**Figure 20:**
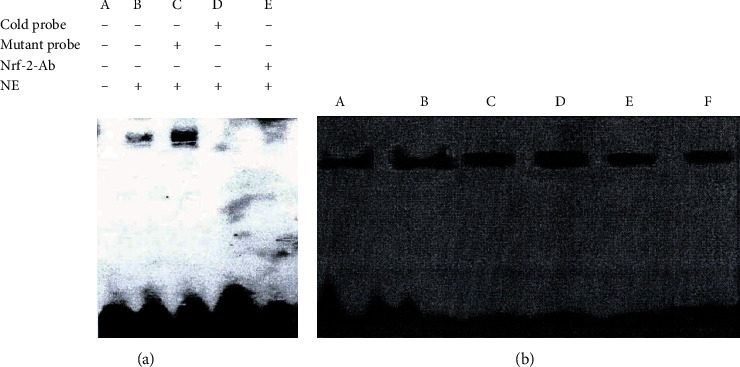
The results of EMSA. (a) Competitive assay. (b) EMSA (A: sham operation group; B: model group; C: FGWYD medium-dose group; D: FGWYD high-dose group; E: FGWYD low-dose group; F: positive control group).

The extracted nucleoprotein and the Nrf2 probe formed an obvious binding zone. The addition of the Nrf2 antibody makes the binding band disappear, indicating that the complex contains Nrf2 protein. A 200-fold concentration of a cold probe can inhibit this binding. If the possible Nrf2 binding element in the cold probe is mutated, the probe loses its binding ability, indicating that it is the Nt-2 binding element that binds to Nrf2 in the probe (Figure 20(a)).

The binding activity of Nrf2-ARE was not obvious in the sham operation group and the model group. The binding activity began to increase in the FGWYD low-, medium-, and high-dose groups and the positive group. Among them, the FGWYD high-dose group was the most significant, and the positive drug group was less (Figure 20(b)).

### 3.9. Effect of FGWYD on the Expression of HO-1 mRNA in VD Rats

**Figure 21:**
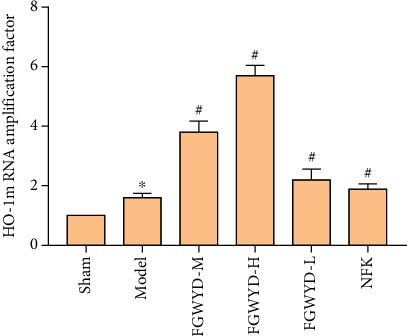
Effect of FGWYD on the expression of HO-1 mRNA in VD rats (compared with the sham operation group, ^∗^*P* < 0.01; compared with the model group, ^#^*P* < 0.05).

Compared with the sham operation group, the expression of HO-1 mRNA in the hippocampus of each group was statistically significantly different (*P* < 0.01). Compared with the model group, the expression of HO-1 mRNA in the FGWYD low-, medium-, and high-dose groups and the positive group was statistically different (*P* < 0.05) (Figure 21).

### 3.10. Effect of FGWYD on the MDA, SOD, GSH-Px, and LPO Contents and T-AOC in VD Rats

**Figure 22:**
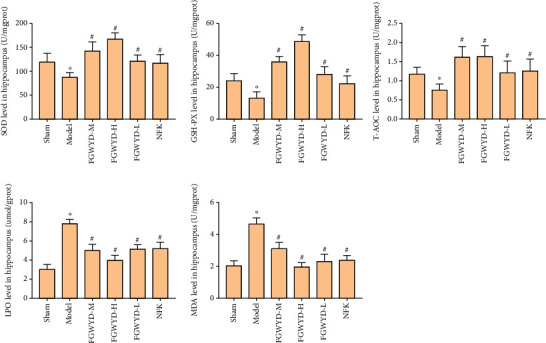
Effect of FGWYD on the MDA, SOD, GSH-Px, LPO, and T-AOC in VD rats (compared with sham operation group, ^∗^*P* < 0.05; compared with model group, ^#^*P* < 0.01).

Compared with sham operation group, the MDA, SOD, GSH-Px, and LPO contents and T-AOC in the model group have statistical significance (*P* < 0.05). Compared with the model group, after drug (FGWYD or piracetam) intervention, the content of MDA and LPO decreased (*P* < 0.01), the content of SOD and GSH-Px increased (*P* < 0.01), and the T-AOC increased (*P* < 0.01) (Figure 22).

**Figure 23:**
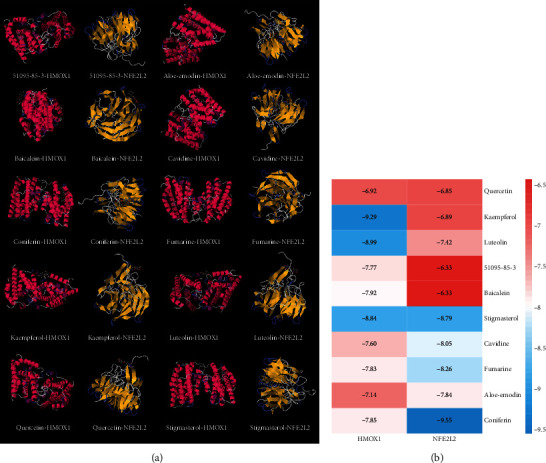
The results of molecular docking ((a) molecular docking mode diagram; (b) binding energy, kcal/mol).

Based on the above comprehensive analysis of network pharmacology and proteomics, we chose oxidative stress as the research direction for further exploration of FGWYD intervention in VD. The main cause of VD is ischemic injury. In the state of ischemia and hypoxia, a large amount of oxygen free radicals will be produced in the brain, which can directly attack the unsaturated fatty acids in the biomembrane phospholipids, causing damage to the brain nerve cells and dementia [37]. Nuclear factor erythroid 2-related factor 2 (Nrf2) interacts with ARE to regulate the encoded antioxidant protein, forming the Nrf2-ARE pathway. This is a new type of antioxidant signaling pathway and the most important endogenous antioxidant stress pathway [38]. A large number of the downstream molecules that it regulates, including HO-1, have multiple functions such as antioxidative stress, regulation of inflammatory damage, and antiapoptosis. Recent studies have shown that abnormal expression of Nrf2 or impaired transcriptional activity is closely related to the occurrence of ischemic encephalopathy [39]. The results of molecular docking showed that the core components of FGWYD can be stably combined with HMOX1 and NFE2L2, suggesting that FGWYD may interfere with VD by interfering with HMOX and NFE2L2 (Figure 23).

Free radical-mediated lipid peroxidation plays an important role in central nervous system diseases such as stroke, neurodegenerative diseases, mental disorders, and nervous system damage [40–43]. The damage caused by free radicals has been running through the process of nerve damage [44]. Free radicals are most likely to attack the double bonds of polyunsaturated acids in the cell membranes of brain cells. Free radicals will continue to damage proteins and nucleic acids, causing cell apoptosis. In nervous system diseases, due to various acute or chronic damages, free radicals will be produced, which will cause a series of cell apoptosis and the destruction of protein and DNA [45]. Current studies have found that the downstream antioxidant system HO-1 regulated by Nrf2-ARE does not contain much in the central nervous system. However, after Nrf2 is activated under prooxidant, inflammatory stimulus, and stress conditions, the expression of HO-1 in glial cells and astrocytes will increase. Studies in degenerative diseases of the central nervous system have shown that the content of HO-1 will also increase significantly, such as Parkinson's disease (PD), multiple sclerosis (MS), and amyotrophic lateral sclerosis (ALS) [46]. As the reduced glutathione contained in brain cells is low, brain cells are more susceptible to oxidative damage [47]. Enhancing the activity of endogenous antioxidant enzymes (such as CAT, GPxs, and SOD) after cerebral ischemia can reduce brain tissue damage [48, 49].

Our study established a rat model of VD caused by AS and used FGWYD for treatment. The results of the study show that FGWYD can activate the Nrf2-ARE pathway to transfer Nrf2 from the cytoplasm to the nucleus and increase its expression in the nucleus. At the same time, Nrf2 is phosphorylated and moved to the nucleus to induce the expression of the HO-1 gene. HO-1 is an important part of brain cells against stress and oxidative damage. It can protect cells from oxidative stress and damage caused by foreign harmful substances.

## 4. Conclusion

The mechanism of FGWYD in the treatment of VD may be related to inflammation, oxidative stress, angiogenesis, and neuronal apoptosis.

## Figures and Tables

**Table 1:** The oral absorbable and pharmacologically active components.

Mol ID	Molecule name	MW	OB (%)	Caco-2	DL	Structure
MOL001506	Supraene	410.8	33.54594	2.08183	0.42161	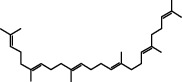
MOL002883	Ethyl oleate (NF)	310.58	32.39739	1.40295	0.19061	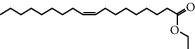
MOL009525	3beta-24S(R)-butyl-5-alkenyl-cholestol	456.88	35.35249	1.36151	0.82221	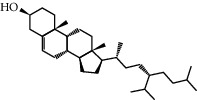
MOL009524	3beta,20(R),5-alkenyl-stigmastol	414.79	36.91391	1.35994	0.75074	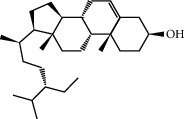
MOL000358	Beta-sitosterol	414.79	36.91391	1.32463	0.75123	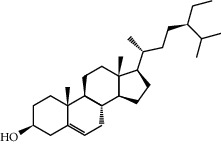
MOL000359	Sitosterol	414.79	36.91391	1.32059	0.7512	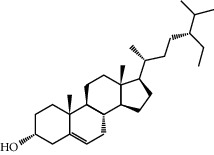
MOL002879	Diop	390.62	43.59333	0.7934	0.39247	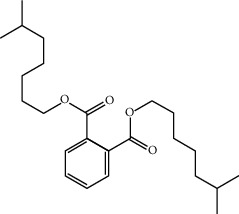
MOL006147	Alizarin-2-methylether	254.25	32.80877	0.61959	0.20971	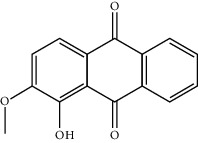
MOL009562	Ohioensin A	372.39	38.13467	0.59512	0.75842	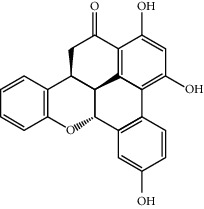
MOL009503	1-Hydroxy-3-methoxy-9,10-anthraquinone	254.25	104.3254	0.58585	0.20915	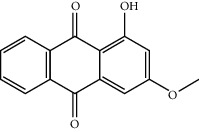
MOL009495	2-Hydroxy-1,5-dimethoxy-6-(methoxymethyl)-9,10-anthraquinone	328.34	95.85174	0.54305	0.37249	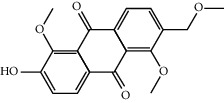
MOL009513	2-Hydroxy-1,8-dimethoxy-7-methoxymethylanthracenequinone	328.34	112.3026	0.45587	0.37164	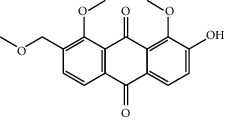
MOL009500	1,6-Dihydroxy-5-methoxy-2-(methoxymethyl)-9,10-anthraquinone	314.31	104.5394	0.36762	0.33917	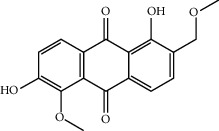
MOL009496	1,5,7-Trihydroxy-6-methoxy-2-methoxymethylanthracenequinone	330.31	80.42295	0.27379	0.37789	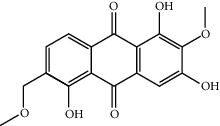
MOL009519	(2R,3S)-(+)-3′,5-Dihydroxy-4,7-dimethoxydihydroflavonol	332.33	77.23782	0.12927	0.33461	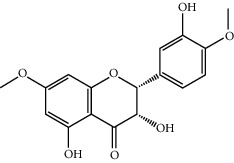
MOL009504	1-Hydroxy-6-hydroxymethylanthracenequinone	254.25	81.76548	-0.03879	0.2115	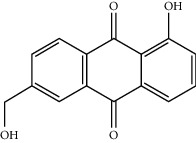
MOL009537	Americanin A	328.34	46.70571	-0.07814	0.34901	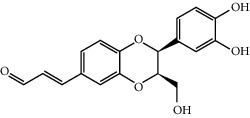
MOL009551	Isoprincepin	494.53	49.12132	-0.18169	0.77375	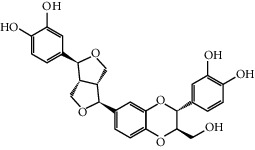
MOL001755	24-Ethylcholest-4-en-3-one	412.77	36.08361	1.4563	0.75703	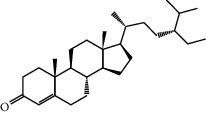
MOL002670	Cavidine	353.45	35.64183	1.0817	0.80513	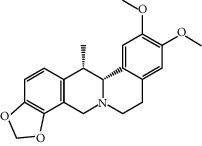
MOL002714	Baicalein	270.25	33.51892	0.63086	0.20888	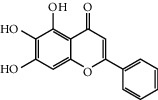
MOL000449	Stigmasterol	412.77	43.82985	1.44458	0.75665	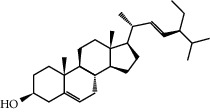
MOL005030	Gondoic acid	310.58	30.70294	1.20473	0.19743	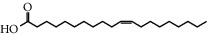
MOL000519	Coniferin	314.41	31.11	0.42439	0.32308	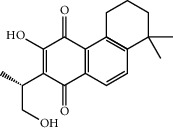
MOL006936	10,13-Eicosadienoic	308.56	39.99355	1.22213	0.20012	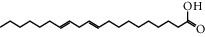
MOL006937	12,13-Epoxy-9-hydroxynonadeca-7,10-dienoic acid	324.51	42.15218	0.17979	0.24248	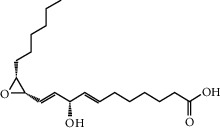
MOL006957	(3S,6S)-3-(Benzyl)-6-(4-hydroxybenzyl)piperazine-2,5-quinone	310.38	46.8889	0.41366	0.26989	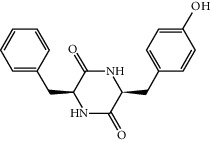
MOL003578	Cycloartenol	426.8	38.68566	1.52617	0.78093	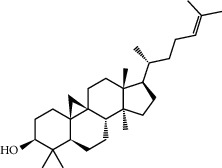
MOL002388	Delphin	303.26	57.7617	0.11969	0.2786	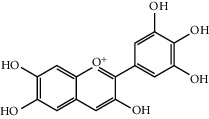
MOL002415	6-Demethyldesoline	453.64	51.87164	-0.25991	0.65822	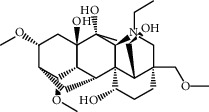
MOL002419	(R)-Norcoclaurine	271.34	82.54295	0.62871	0.20872	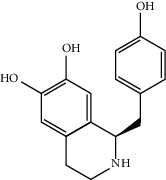
MOL002397	Karakoline	377.58	51.7309	0.32469	0.73447	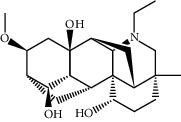
MOL002421	Ignavine	449.59	84.07948	-0.07071	0.24798	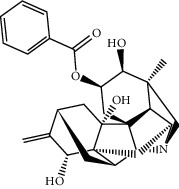
MOL002422	Isotalatizidine	407.61	50.82414	-0.10596	0.73291	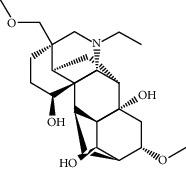
MOL002395	Deoxyandrographolide	334.5	56.3041	0.181	0.31451	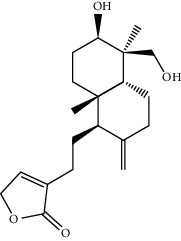
MOL002410	Benzoylnapelline	463.67	34.0565	0.19203	0.52933	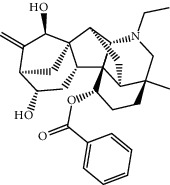
MOL002416	Deoxyaconitine	629.82	30.95922	-0.2338	0.24469	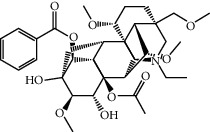
MOL002434	Carnosifloside I	456.78	38.15575	0.2846	0.79654	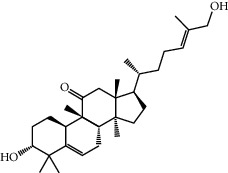
MOL000538	Hypaconitine	615.79	31.38846	-0.3365	0.26085	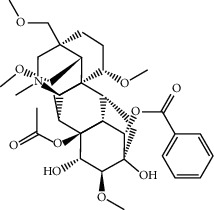
MOL002211	11,14-Eicosadienoic acid	308.56	39.99355	1.21793	0.20044	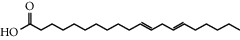
MOL002401	Neokadsuranic acid B	452.74	43.09829	0.68515	0.85195	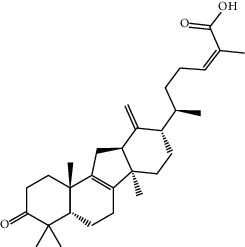
MOL002392	Deltoin	328.39	46.69281	0.55431	0.36837	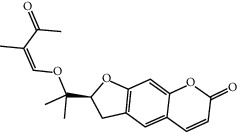
MOL002398	Karanjin	292.3	69.55687	1.22452	0.33616	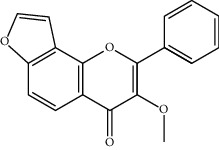
MOL002464	1-Monolinolein	354.59	37.17663	0.31862	0.30249	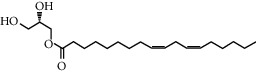
MOL002501	[(1S)-3-[(E)-but-2-enyl]-2-methyl-4-oxo-1-cyclopent-2-enyl] (1R,3R)-3-[(E)-3-methoxy-2-methyl-3-oxoprop-1-enyl]-2,2-dimethylcyclopropane-1-carboxylate	360.49	62.51583	0.36515	0.30983	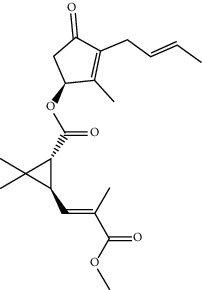
MOL002514	Sexangularetin	316.28	62.85792	0.31432	0.2968	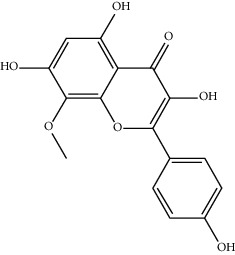
MOL001736	(-)-Taxifolin	304.27	60.50622	-0.24278	0.27342	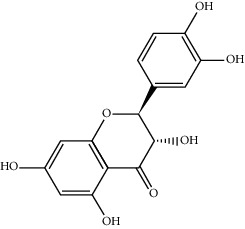
MOL000492	(+)-Catechin	290.29	54.82643	-0.03424	0.24164	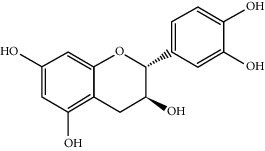
MOL000073	ent-Epicatechin	290.29	48.95984	0.01948	0.24162	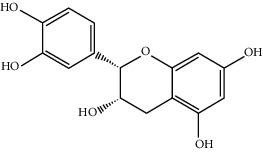
MOL004576	Taxifolin	304.27	57.84156	-0.22844	0.27345	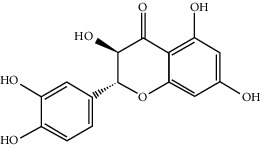
MOL011169	Peroxyergosterol	428.72	44.39152	0.86327	0.82	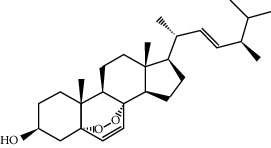
MOL001510	24-Epicampesterol	400.76	37.57682	1.43482	0.71413	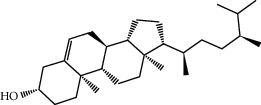
MOL001645	Linoleyl acetate	308.56	42.10077	1.35826	0.19845	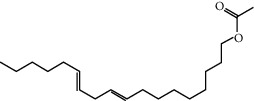
MOL001771	Poriferast-5-en-3beta-ol	414.79	36.91391	1.45001	0.75034	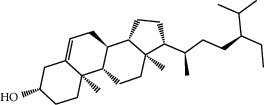
MOL001792	Liquiritigenin	256.27	32.76272	0.50823	0.18316	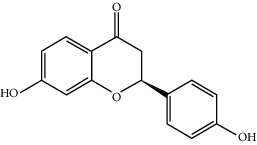
MOL003044	Chryseriol	300.28	35.85089	0.39361	0.27415	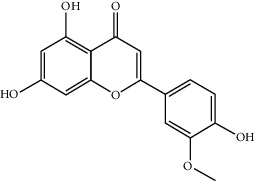
MOL003542	8-Isopentenyl-kaempferol	354.38	38.04434	0.53297	0.3948	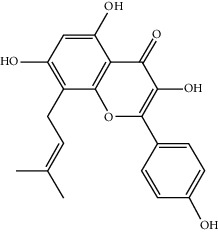
MOL000422	Kaempferol	286.25	41.88225	0.26096	0.24066	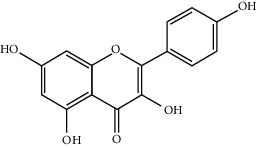
MOL004367	Olivil	376.44	62.2286	-0.1612	0.40642	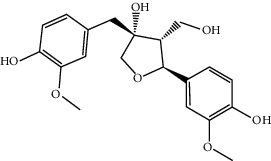
MOL004373	Anhydroicaritin	368.41	45.41193	0.72306	0.43786	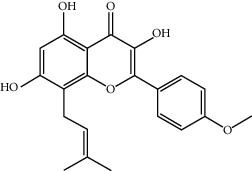
MOL004380	C-Homoerythrinan, 1,6-didehydro-3,15,16-trimethoxy-, (3.beta.)-	329.48	39.13993	1.01828	0.49461	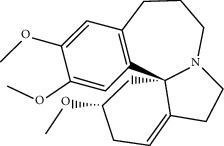
MOL004382	Yinyanghuo A	420.49	56.95738	0.37565	0.76747	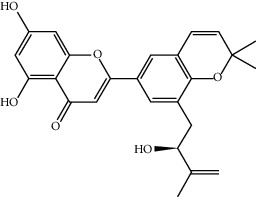
MOL004384	Yinyanghuo C	336.36	45.672	0.74533	0.50155	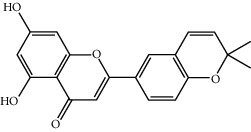
MOL004386	Yinyanghuo E	352.36	51.63213	0.50883	0.5474	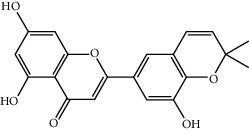
MOL004388	6-Hydroxy-11,12-dimethoxy-2,2-dimethyl-1,8-dioxo-2,3,4,8-tetrahydro-1H-isochromeno[3,4-h]isoquinolin-2-ium	370.41	60.64151	0.34258	0.65693	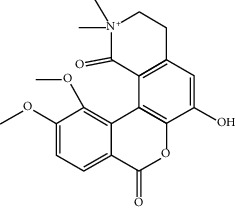
MOL004391	8-(3-Methylbut-2-enyl)-2-phenyl-chromone	290.38	48.5445	1.52596	0.25066	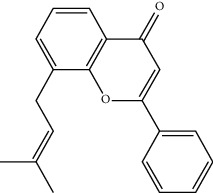
MOL004396	1,2-bis(4-Hydroxy-3-methoxyphenyl)propan-1,3-diol	320.37	52.31425	0.0015	0.22066	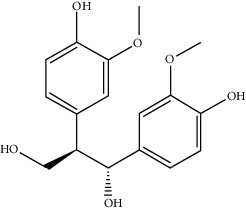
MOL000006	Luteolin	286.25	36.16263	0.185	0.24552	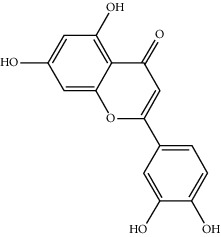
MOL000622	Magnograndiolide	266.37	63.70888	0.02344	0.18833	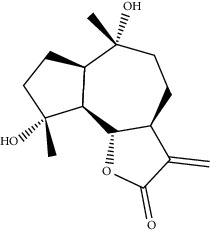
MOL000098	Quercetin	302.25	46.43335	0.04842	0.27525	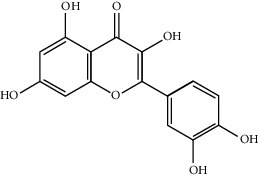
MOL003576	(1R,3aS,4R,6aS)-1,4-bis(3,4-Dimethoxyphenyl)-1,3,3a,4,6,6a-hexahydrofuro[4,3-c]furan	386.48	52.34558	0.83015	0.62031	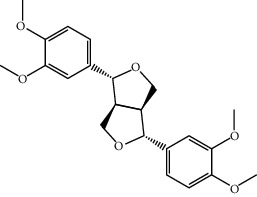
MOL001494	Mandenol	308.56	41.9962	1.45585	0.19321	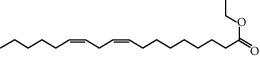
MOL003648	Inermin	284.28	65.83093	0.91157	0.53754	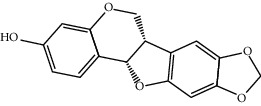
MOL004492	Chrysanthemaxanthin	584.96	38.72398	0.50972	0.58352	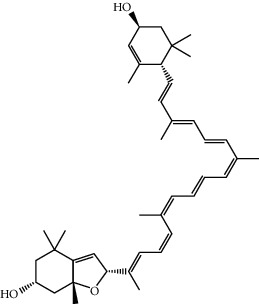
MOL005308	Aposiopolamine	271.34	66.64691	0.65617	0.21999	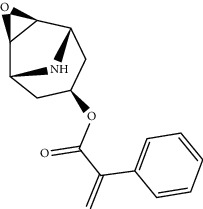
MOL005314	Celabenzine	379.55	101.8826	0.77185	0.48772	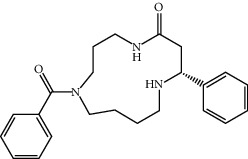
MOL005317	Deoxyharringtonine	515.66	39.27444	0.18714	0.8116	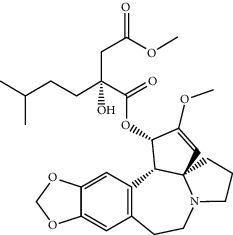
MOL005318	Dianthramine	289.26	40.44641	-0.22511	0.19676	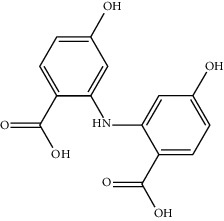
MOL005320	Arachidonate	304.52	45.57325	1.26865	0.20491	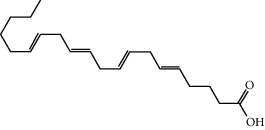
MOL005321	Frutinone A	264.24	65.90373	0.88838	0.34184	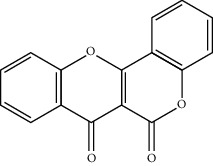
MOL005348	Ginsenoside-Rh4	458.8	31.11215	0.49755	0.77829	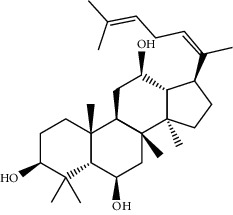
MOL005356	Girinimbin	263.36	61.2153	1.72097	0.31484	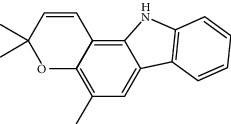
MOL005357	Gomisin B	514.62	31.99042	0.60183	0.82858	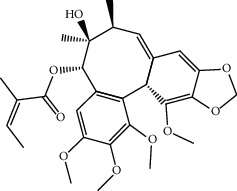
MOL005360	Malkangunin	432.56	57.71384	0.21673	0.62642	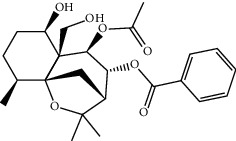
MOL005376	Panaxadiol	460.82	33.08796	0.82469	0.79404	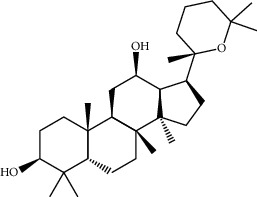
MOL005384	Suchilactone	368.41	57.51882	0.82023	0.55573	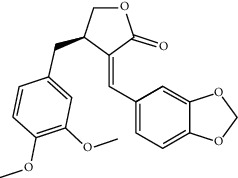
MOL005399	Alexandrin	414.79	36.91391	1.30404	0.75268	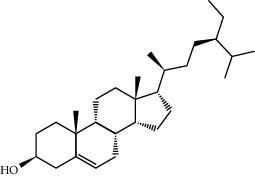
MOL005401	Ginsenoside Rg5	442.8	39.56307	0.87742	0.78506	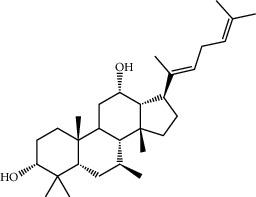
MOL000787	Fumarine	353.4	59.2625	0.56266	0.82694	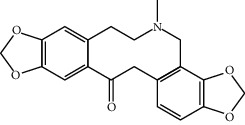

**Table 2:** The oligonucleotide sequences.

Gene	Method	Forward sequence	Reverse sequence
Nrf2/ARE oligonucleotide	EMSA	TTTATGCTGTGTCATGGTT	AACCATACACAGCATAAAA
Nrf2/mutARE oligonucleotide	EMSA	TTTTATGCAGACACATGGTT	AACCATACTGTCTATAAAA

**Table 3:** The primer.

Gene	Sequence
HO-1	F: AGAGGGTGATAGAAGAGGCCAA
	P: GTGTAAGGACCCATCGGAGAAG
*β*-Actin	F: AGGGGCCGOACTCGTCATACT
	P: GGCGGCACCACCATGTACCCT

**Table 4:** Sample marking information.

Sample	Label
CK1	113
CK2	114
HD1	115
HD2	116
M1	117
M2	118
W1	119
W2	121

**Table 5:** Clusters of FGWYD-VD PPI network.

Cluster	Score	Nodes	Edges	Targets and genes
1	44.214	57	1238	PLAU, MPO, CCL2, CXCL8, TGFB1, NOS2, MAPK14, SERPINE1, CAT, CASP3, BDNF, CRP, NGF, CDKN1A, CXCL10, SPP1, TP53, MYC, IL1B, CCNB1, HMOX1, RELA, AKT1, FOS, MMP9, HIF1A, TNFRSF1A, CD40, JUN, MAPK8, MMP1, STAT1, ICAM1, VCAM1, PTGS2, EGFR, BCL2L1, CASP9, MMP2, MAPK1, IL10, PPARG, IL6, MDM2, MCL1, IL2, AR, IFNG, IL4, CD40LG, APOE, ACE, TNF, MMP3, EGF, CASP8, VEGFA
2	13.778	46	310	NOS3, HSPA5, IKBKB, SP1, CAV1, F3, GJA1, SELE, IL1A, AGER, GSK3B, CDK2, CHEK1, PTEN, NCF1, CCNA2, PARP1, KDR, CXCL2, CHEK2, HSPB1, HSPB2, HSPB3, CCND1, ESR1, HSP90AA1, RUNX2, RB1, CDK1, CDK4, NFKBIA, IGFBP3, IRF1, PGR, ERBB2, CASP7, BIRC5, ADAM17, XIAP, APP, AHR, IGF2, MET, NR3C1, NFE2L2, RAF1
3	12.533	16	94	DPP4, DRD2, OPRM1, PTGER3, HTR1B, ADRA2A, HTR1A, APOB, ADCY2, CHRM4, CXCL11, OPRD1, GAL, ADRA2C, CHRM2, ADRA2B
4	7.6	21	76	ADRA1B, NCOA2, PRKCA, RXRA, ADRA1D, MAOB, NCOA1, CHRM3, CYP1A1, CHRM1, HTR2A, MAOA, CHRM5, GRIN1, HTR3A, PPP3CA, VIP, HRH1, ADRA1A, SNCA, HTR2C
5	5.429	8	19	GCLM, GCLC, GSTP1, GSS, PON1, GSTM1, GSTM2, AKR1B1
6	4.571	8	16	GRIA2, SNCB, GABRA6, GABRG3, GABRE, CLDN4, MT3, GABRA3
7	4.5	9	18	SLC6A3, SYP, SLC6A4, CALM1, GRIN2A, CHRNA4, SLC6A2, CRH, CHAT
8	4.286	8	15	ADRB1, ADRB2, PRKCD, GRIN2B, ACHE, PRKCB, DRD1, DRD5
9	3.714	8	13	SQSTM1, MAPT, CTSD, BACE1, PSEN1, BCL2, TFRC, CHUK
10	3.5	5	7	AKR1C3, CYP1B1, SULT1E1, AKR1C1, NR1I2
11	3	3	3	F2, PLAT, THBD
12	3	3	3	COX5A, F7, F10
13	2.8	6	7	GFAP, KEAP1, MBP, S100B, NQO1, SOD1

**Table 6:** The differential protein between the sham operation group and the model group.

Protein TD	Description	Regulated
F1LPB4	Protein Akap9	Up
D4A050	Protein Tbcld32	Up
F1LUM5	Tubulin alpha chain	Up
Q63450	Calcium/calmodulin-dependent protein kinase type 1	Up
A0A0G2K5Q2	Crooked neck-like protein 1	Up
R9PXS3	Transcription elongation factor, mitochondrial	Up
A0A0G2K1C7	Protein RGD1566386	Up
Q91ZW6	Trimethyllysine dioxygenase, mitochondrial	Up
F1M3H3	Protein Frasl	Up
P28470	Calcineurin subunit B type 2	Up
M0RRJ7	Complement C3	Up
Q5U2R9	Protein Scfd2	Up
B0BNJ9	RCG44002, isoform CRA a	Up
Q5HZD9	LOC100125377 protein	Up
D4Al l7	SID 1 transmembrane family member 1	Up
M0RCK7	G-protein-coupled receptor 1	Up
Q3SWT7	Nuclear receptor binding protein	Up
F1LPTO	Gap junction protein	Up
D3ZA65	Protein Stk36	Up
M0R660	Glyceraldehyde-3-phosphate dehydrogenase	Up
F1LTH9	Protein Wrn	Up
D4A3T5	Protein C 1 q13	Up
M0RBJ0	Guanine nucleotide-binding protein subunit gamma	Up
D3ZW33	Protein Bach2	Down
D3ZTJ6	Protein Tpcn2	Down
Q5M7T1	Probable cytosolic iron-sulfur protein assembly protein CIAO1	Down
QSXIR9	Ubiquitin-associated domain-containing protein 1	Down
Q510K8	28S ribosomal protein S7, mitochondrial	Down
Q91V33	KHdomain-containing, RNA-binding signal transduction-associated protein 1	Down
M0R5Q3	Protein Ranbp3	Down
A0A0G2K719	Protein Ddx3x	Down
D3ZE71	Protein Faap24	Down
Q5XIMS	Protein CDV3 homolog	Down
D3ZWV2	Glyceraldehyde-3-phosphate dehydrogenase	Down
D4A4U3	Protein Mdpl	Down
F1LWK7	Protein Ablim 1 (fragment)	Down
F1MlA6	Protein T,OC681355	Down
D3ZEL3	Protein TmcoSb	Down
G3V6S6	Protein Suv39h1l1	Down
A0A0G2K475	Protein Brip 1	Down

**Table 7:** the differential protein between the FGWYD group and the model group.

Protein ID	Description	Regulated
A0A0G2K475	Protein Brip 1	Up
Q5XIC2	Evolutionarily conserved signaling intermediate in Toll pathway, mitochondrial	Up
D3ZWV2	Glyceraldehyde-3-phosphate dehydrogenase	Up
G3V6S6	Protein Suv39h111	Up
D3ZF71	Protein Faap24	Up
D3ZFL3	Protein TmcoSb	Up
F1LWK7	Protein Ablim 1 (fragment)	Up
Q5M7T1	Probable cytosolic iron-sulfur protein assembly protein CIAO1	Up
A0A096MK30	Moesin	Up
A0A0G2JVA8	Protein Kb15	Up
P62329	Thymosin beta-4	Up
D4A050	Protein Tbcl d32	Down
Q5U2R9	Protein Scfd2	Down
MORBJ7	Complement C3	Down
D4A117	SID 1 transmembrane family member 1	Down
A0A0G2K5Q2	Crooked neck-like protein 1	Down
F1M3H3	Protein Frasl	Down
F1LUM5	Tubulin alpha chain	Down
F1LPB4	Protein Akap9	Down
Q5XIG9	Mitochondrial protein 18 kDa OS	UP
A0A0G2K475	Protein Brip 1	UP
D4ACK7	Protein Cnnm3	UP
P62329	Thymosin beta-4	UP
F1LNC3	PH domain leucine-rich repeat protein phosphatase 1	UP
D4A050	Protein Tbcl d32	Down
F1LPB4	Protein Akap9	Down

## Data Availability

The data used to support the findings of this study are included within the article and the supplementary information files.

## Supplementary Materials

Supplementary 1 Table S1: potential targets for FGWYD.

Supplementary 2 Table S2: VD genes.

Supplementary 3 Table S3: biological processes of the FGWYD-VD PPI network.

Supplementary 4 Table S4: signaling pathway of the FGWYD-VD PPI network.

Supplementary 5 Table S5: proteomics data and other rat proteins.

Supplementary 6 Table S6: bioinformatics analysis of differential expression of the protein-other rat protein PPI network.

Supplementary 7 Figure S1: the results of HPLC: (a) reference solution; (b) FGWYD solution.
