# Supplementary material for: Systems Biology and Chemoinformatics-Based Strategies to Explore the Biological Mechanism of Fugui Wenyang Decoction in Treating Vascular Dementia Rats
Source: Oxid Med Cell Longev. 2021 Oct 7;2021:6693955. doi: 10.1155/2021/6693955 (PMC8517630; doi:10.1155/2021/6693955)
Supplement: Supplementary 1 — Table S1: potential targets for FGWYD. [file 6693955.f1.pdf]

Table S1 Potential Targets of FGWYD

Compounds	Compounds Targets
(-)-catechin	PTGS1
(-)-catechin	ESR1
(-)-catechin	PTGS2
(-)-catechin	NCOA2
(-)-catechin	FASN
(-)-catechin	PPARG
(-)-catechin	KLF7
(-)-catechin	CALM1
(-)-catechin	CALM2
(-)-catechin	CALM3
(-)-taxifolin	PTGS1
(-)-taxifolin	PTGS2
(-)-taxifolin	PIK3CG
(+)-catechin	PTGS1
(+)-catechin	ESR1
(+)-catechin	PTGS2
(+)-catechin	HSP90AA1
(+)-catechin	NCOA2
(+)-catechin	RXRA
(+)-catechin	CAT
(+)-catechin	HAS2
(+)-catechin	CALM1
(+)-catechin	CALM2
(+)-catechin	CALM3
10,13-eicosadienoi	PTGS1
10,13-eicosadienoi	NCOA2
11,14-eicosadienoi	NCOA2
24-epicampesterol	PGR
24-epicampesterol	NCOA2
24-Ethylcholest-4-	PGR
24-Ethylcholest-4-	NR3C2
3beta,20(R),5-alke	PGR
3beta-24S(R)-buty	PGR
51095-85-3	PTGS1
51095-85-3	DRD1
51095-85-3	CHRM3
51095-85-3	KCNH2
51095-85-3	CHRM1
51095-85-3	AR
51095-85-3	DRD5
51095-85-3	ADRB1
51095-85-3	SCN5A
51095-85-3	CHRM5
51095-85-3	PTGS2
51095-85-3	ADRA2A
51095-85-3	HTR1A

51095-85-3	HTR3A
51095-85-3	CHRM4
51095-85-3	OPRD1
51095-85-3	PDE3A
51095-85-3	HRH1
51095-85-3	HTR2A
51095-85-3	SLC6A2
51095-85-3	ADRA1A
51095-85-3	GABRA3
51095-85-3	HTR2C
51095-85-3	CHRM2
51095-85-3	ADRA2B
51095-85-3	ADRA1B
51095-85-3	SLC6A3
51095-85-3	ADRB2
51095-85-3	ADRA1D
51095-85-3	CHRNA2
51095-85-3	SLC6A4
51095-85-3	DRD2
51095-85-3	OPRM1
51095-85-3	GABRA1
51095-85-3	HTR1B
51095-85-3	CHRNA7
51095-85-3	GABRG3
51095-85-3	GABRE
6-methylgingediac	ESR1
6-methylgingediac	F10
6-methylgingediac	PTGS2
6-methylgingediac	CALM1
6-methylgingediac	CALM2
6-methylgingediac	CALM3
8-(3-methylbut-2-e	NOS2
8-(3-methylbut-2-e	PTGS1
8-(3-methylbut-2-e	CHRM3
8-(3-methylbut-2-e	F2
8-(3-methylbut-2-e	CHRM1
8-(3-methylbut-2-e	ESR1
8-(3-methylbut-2-e	AR
8-(3-methylbut-2-e	ADRB1
8-(3-methylbut-2-e	SCN5A
8-(3-methylbut-2-e	PPARG
8-(3-methylbut-2-e	PTGS2
8-(3-methylbut-2-e	NOS3
8-(3-methylbut-2-e	RXRA
8-(3-methylbut-2-e	ACHE
8-(3-methylbut-2-e	PDE3A
8-(3-methylbut-2-e	ADRA1B
8-(3-methylbut-2-e	SLC6A3

8-(3-methylbut-2-e	ADRB2
8-(3-methylbut-2-e	ADRA1D
8-(3-methylbut-2-e	SLC6A4
8-(3-methylbut-2-e	ESR2
8-(3-methylbut-2-e	GABRA1
8-(3-methylbut-2-e	DPP4
8-(3-methylbut-2-e	MAPK14
8-(3-methylbut-2-e	GSK3B
8-(3-methylbut-2-e	CDK2
8-(3-methylbut-2-e	CHEK1
8-(3-methylbut-2-e	PIM1
8-(3-methylbut-2-e	CCNA2
8-(3-methylbut-2-e	F10
8-(3-methylbut-2-e	TOP2A
8-Isopentenyl-kaer	CDK2
8-Isopentenyl-kaer	CHEK1
8-Isopentenyl-kaer	PRSS1
8-Isopentenyl-kaer	PIM1
8-Isopentenyl-kaer	PTGS1
8-Isopentenyl-kaer	SCN5A
8-Isopentenyl-kaer	F7
8-Isopentenyl-kaer	KDR
8-Isopentenyl-kaer	RXRA
8-Isopentenyl-kaer	MAPK14
8-Isopentenyl-kaer	CCNA2
8-Isopentenyl-kaer	KCNMA1
8-Isopentenyl-kaer	NCOA2
8-Isopentenyl-kaer	GABRA2
8-Isopentenyl-kaer	GABRA1
8-Isopentenyl-kaer	PDE3A
8-Isopentenyl-kaer	KCNH2
8-Isopentenyl-kaer	PYGM
8-Isopentenyl-kaer	CALM1
8-Isopentenyl-kaer	CALM2
8-Isopentenyl-kaer	CALM3
Alexandrin	PGR
Alizarin-2-methyle	PTGS1
Alizarin-2-methyle	ESR1
Alizarin-2-methyle	PTGS2
Alizarin-2-methyle	RXRA
Alizarin-2-methyle	PDE3A
Alizarin-2-methyle	SLC6A4
Alizarin-2-methyle	GABRA1
Alizarin-2-methyle	PIK3CG
Alizarin-2-methyle	CHRNA7
Alizarin-2-methyle	NCOA2
Alizarin-2-methyle	PKIA
Aloe-emodin	PTGS1

Aloe-emodin	PTGS2
Aloe-emodin	PIK3CG
Aloe-emodin	NCOA2
Aloe-emodin	PKIA
Aloe-emodin	AKR1B1
Aloe-emodin	IGHG1
Aloe-emodin	CDKN1A
Aloe-emodin	EIF6
Aloe-emodin	BAX
Aloe-emodin	TNF
Aloe-emodin	CASP3
Aloe-emodin	TP53
Aloe-emodin	FASN
Aloe-emodin	PRKCA
Aloe-emodin	PRKCE
Aloe-emodin	CDK1
Aloe-emodin	PCNA
Aloe-emodin	MYC
Aloe-emodin	IL1B
Aloe-emodin	PRKCD
Aloe-emodin	CCNB1
Americanin A	ESR1
Americanin A	SCN5A
Americanin A	PTGS2
Americanin A	DPP4
Americanin A	GSK3B
Americanin A	CDK2
Americanin A	PRSS1
Americanin A	PIM1
Americanin A	CCNA2
Anhydroicaritin	NOS2
Anhydroicaritin	PTGS1
Anhydroicaritin	CHRM3
Anhydroicaritin	F2
Anhydroicaritin	KCNH2
Anhydroicaritin	CHRM1
Anhydroicaritin	ESR1
Anhydroicaritin	AR
Anhydroicaritin	SCN5A
Anhydroicaritin	PPARG
Anhydroicaritin	F10
Anhydroicaritin	CHRM5
Anhydroicaritin	PTGS2
Anhydroicaritin	NOS3
Anhydroicaritin	RXRA
Anhydroicaritin	ACHE
Anhydroicaritin	ADRA1B
Anhydroicaritin	ADRB2

Anhydroicaritin	ESR2
Anhydroicaritin	DPP4
Anhydroicaritin	MAPK14
Anhydroicaritin	GSK3B
Anhydroicaritin	CDK2
Anhydroicaritin	CHEK1
Anhydroicaritin	RXRB
Anhydroicaritin	PRSS1
Anhydroicaritin	PIM1
Anhydroicaritin	NCOA2
Anhydroicaritin	F7
Anhydroicaritin	KDR
Anhydroicaritin	TOP2A
Anhydroicaritin	CCNA2
Anhydroicaritin	NCOA1
Anhydroicaritin	KCNMA1
Anhydroicaritin	IGHG1
Anhydroicaritin	CALM1
Anhydroicaritin	CALM2
Anhydroicaritin	CALM3
Aposiopolamine	CHRM3
Aposiopolamine	CHRM1
Aposiopolamine	SLC6A2
Aposiopolamine	SLC6A3
Aposiopolamine	ADRB2
Aposiopolamine	SLC6A4
Aposiopolamine	GABRA1
Aposiopolamine	DPP4
Arachidonate	PTGS1
Arachidonate	PTGS2
Arachidonate	RXRG
Arachidonate	NCOA2
Baicalein	PTGS1
Baicalein	AR
Baicalein	PTGS2
Baicalein	DPP4
Baicalein	PIK3CG
Baicalein	PDE3A
Baicalein	PRSS1
Baicalein	NCOA2
Baicalein	NCOA1
Baicalein	RELA
Baicalein	AKT1
Baicalein	VEGFA
Baicalein	BCL2
Baicalein	FOS
Baicalein	BAX
Baicalein	MMP9

Baicalein	CASP3
Baicalein	TP53
Baicalein	HIF1A
Baicalein	FOSL1
Baicalein	FOSL2
Baicalein	CDK1
Baicalein	CCNB1
Baicalein	MPO
Baicalein	AHR
Baicalein	IGF2
Baicalein	ALOX12
Baicalein	NFATC1
Baicalein	TDRD7
Baicalein	EGLN1
Baicalein	NOX5
Baicalein	FABP5
Baicalein	APOD
Baicalein	CALM1
Baicalein	CALM2
Baicalein	CALM3
Beta-sitosterol	PGR
Beta-sitosterol	NCOA2
Beta-sitosterol	NR3C2
Cavidine	PTGS1
Cavidine	CHRM3
Cavidine	KCNH2
Cavidine	CHRM1
Cavidine	ADRB1
Cavidine	SCN5A
Cavidine	F10
Cavidine	CHRM5
Cavidine	PTGS2
Cavidine	HTR3A
Cavidine	ADRA2C
Cavidine	CHRM4
Cavidine	RXRA
Cavidine	OPRD1
Cavidine	HTR2A
Cavidine	HTR2C
Cavidine	ADRA1B
Cavidine	ADRB2
Cavidine	ADRA1D
Cavidine	TOP2A
Cavidine	OPRM1
Cavidine	RXRB
Cavidine	DRD1
Cavidine	SLC6A4
Cavidine	F7

Cavidine	PDE10A
Cavidine	CALM1
Cavidine	CALM2
Cavidine	CALM3
Chryseriol	NOS2
Chryseriol	PTGS1
Chryseriol	ESR1
Chryseriol	AR
Chryseriol	PPARG
Chryseriol	PTGS2
Chryseriol	DPP4
Chryseriol	MAPK14
Chryseriol	GSK3B
Chryseriol	CDK2
Chryseriol	PIK3CG
Chryseriol	CHEK1
Chryseriol	PRSS1
Chryseriol	NCOA2
Chryseriol	NCOA1
Chryseriol	CALM1
Chryseriol	CALM2
Chryseriol	CALM3
Coniferin	CHRM3
Coniferin	CHRM1
Coniferin	ESR1
Coniferin	AR
Coniferin	SCN5A
Coniferin	PPARG
Coniferin	PTGS2
Coniferin	CA2
Coniferin	PDE3A
Coniferin	ADRA1B
Coniferin	ADRB2
Coniferin	ADRA1D
Coniferin	TOP2A
Coniferin	OPRM1
Coniferin	CDK2
Coniferin	CHRNA7
Coniferin	IGHG1
Coniferin	PIM1
Coniferin	CCNA2
Coniferin	NCOA2
Coniferin	NCOA1
Cycloartenol	NR3C2
Delphin	PTGS1
Delphin	PTGS2
Delphin	CA2
Delphin	PIK3CG

Delphin	NCOA2
Deltoin	PTGS1
Deltoin	F2
Deltoin	SCN5A
Deltoin	F10
Deltoin	PTGS2
Deltoin	ACHE
Deltoin	ADRA1B
Deltoin	ADRB2
Deltoin	DPP4
Deltoin	PRSS1
Deltoin	CALM1
Deltoin	CALM2
Deltoin	CALM3
Deoxyandrographo	PTGS2
Deoxyandrographo	PGR
Deoxyandrographo	NCOA2
Deoxyandrographo	NCOA1
Deoxyharringtonin	AR
Deoxyharringtonin	NR3C2
Dianthramine	PTGS1
Dianthramine	PTGS2
Diop	SCN5A
Diop	ADRB2
Diop	CHRM3
ent-Epicatechin	PTGS1
ent-Epicatechin	ESR1
ent-Epicatechin	PTGS2
Ethyl oleate (NF)	NCOA2
Eudesmin	CHRM3
Eudesmin	KCNH2
Eudesmin	SCN5A
Eudesmin	F10
Eudesmin	PTGS2
Eudesmin	ADRA1B
Eudesmin	ADRA1D
Eudesmin	NCOA2
Eudesmin	CALM1
Eudesmin	CALM2
Eudesmin	CALM3
Eupatin	NOS2
Eupatin	AR
Eupatin	F10
Eupatin	PTGS2
Eupatin	F7
Eupatin	TOP2A
Eupatin	ESR2
Eupatin	DPP4

Eupatin	PRSS1
Eupatin	NCOA2
Eupatin	F2
Eupatin	SCN5A
Eupatin	KDR
Eupatin	PPARD
Eupatin	CALM1
Eupatin	CALM2
Eupatin	CALM3
Frutinone A	PTGS1
Frutinone A	F2
Frutinone A	AR
Frutinone A	SCN5A
Frutinone A	PPARG
Frutinone A	PTGS2
Frutinone A	RXRA
Frutinone A	PDE3A
Frutinone A	ADRB2
Frutinone A	GABRA1
Frutinone A	DPP4
Frutinone A	PIK3CG
Frutinone A	CHRNA7
Frutinone A	ACHE
Fumarine	PTGS1
Fumarine	CHRM3
Fumarine	KCNH2
Fumarine	CHRM1
Fumarine	SCN5A
Fumarine	F10
Fumarine	CHRM5
Fumarine	PTGS2
Fumarine	HTR3A
Fumarine	F7
Fumarine	CHRM4
Fumarine	OPRD1
Fumarine	HTR2A
Fumarine	ADRA1B
Fumarine	ADRB2
Fumarine	ADRA1D
Fumarine	OPRM1
Fumarine	SLC6A4
Fumarine	CACNA1S
Fumarine	PDE3A
Fumarine	SLC6A3
Fumarine	TOP2A
Fumarine	DRD1
Fumarine	KDR
Fumarine	CALM1

Fumarine	CALM2
Fumarine	CALM3
Ginsenoside Rh4	NR3C2
Ginsenoside Rh4	NCOA2
Girinimbin	PTGS1
Girinimbin	SCN5A
Girinimbin	PTGS2
Girinimbin	RXRA
Girinimbin	ADRB2
Girinimbin	GABRA1
Girinimbin	PIK3CG
Girinimbin	CHRNA7
Girinimbin	NCOA2
Gondoic acid	PTGS1
Gondoic acid	NCOA2
Inermin	PTGS1
Inermin	CHRM3
Inermin	SCN5A
Inermin	PTGS2
Inermin	HTR3A
Inermin	RXRA
Inermin	ADRA1B
Inermin	ADRB2
Inermin	ADRA1D
Inermin	SLC6A4
Inermin	PIK3CG
Inermin	CHRNA7
Inermin	IGHG1
Inermin	PRSS1
Inermin	NCOA1
Inermin	CALM1
Inermin	CALM2
Inermin	CALM3
Isoprincepin	ACHE
Isoprincepin	PRSS1
Kaempferol	NOS2
Kaempferol	PTGS1
Kaempferol	AR
Kaempferol	PPARG
Kaempferol	PTGS2
Kaempferol	HSP90AA1
Kaempferol	PIK3CG
Kaempferol	NCOA2
Kaempferol	DPP4
Kaempferol	PRSS1
Kaempferol	PGR
Kaempferol	F2
Kaempferol	CHRM1

Kaempferol	NOS3
Kaempferol	GABRA2
Kaempferol	ACHE
Kaempferol	SLC6A2
Kaempferol	CHRM2
Kaempferol	ADRA1B
Kaempferol	GABRA1
Kaempferol	TOP2A
Kaempferol	F7
Kaempferol	RELA
Kaempferol	IKBKB
Kaempferol	AKT1
Kaempferol	BCL2
Kaempferol	BAX
Kaempferol	TNF
Kaempferol	JUN
Kaempferol	AHSA1
Kaempferol	CASP3
Kaempferol	MAPK8
Kaempferol	XDH
Kaempferol	MMP1
Kaempferol	STAT1
Kaempferol	CDK1
Kaempferol	HMOX1
Kaempferol	CYP3A4
Kaempferol	CYP1A2
Kaempferol	CYP1A1
Kaempferol	ICAM1
Kaempferol	SELE
Kaempferol	VCAM1
Kaempferol	NR1I2
Kaempferol	CYP1B1
Kaempferol	ALOX5
Kaempferol	HAS2
Kaempferol	GSTP1
Kaempferol	AHR
Kaempferol	PSMD3
Kaempferol	SLC2A4
Kaempferol	NR1I3
Kaempferol	INSR
Kaempferol	DIO1
Kaempferol	PPP3CA
Kaempferol	GSTM1
Kaempferol	GSTM2
Kaempferol	AKR1C3
Kaempferol	SLPI
Kaempferol	CALM1
Kaempferol	CALM2

Kaempferol	CALM3
Karanjin	PTGS1
Karanjin	ESR1
Karanjin	PTGS2
Karanjin	PIK3CG
Karanjin	CHEK1
Linoleyl acetate	PTGS1
Linoleyl acetate	PTGS2
Linoleyl acetate	NCOA2
Linoleyl acetate	RXRA
Liquiritigenin	PTGS1
Liquiritigenin	ESR1
Liquiritigenin	PTGS2
Liquiritigenin	RXRA
Liquiritigenin	ADRB2
Liquiritigenin	PIK3CG
Liquiritigenin	MAOB
Liquiritigenin	SLC6A4
Liquiritigenin	PKIA
Luteolin	PTGS1
Luteolin	AR
Luteolin	PTGS2
Luteolin	HSP90AA1
Luteolin	PRSS1
Luteolin	NCOA2
Luteolin	DPP4
Luteolin	PIK3CG
Luteolin	RELA
Luteolin	EGFR
Luteolin	AKT1
Luteolin	VEGFA
Luteolin	CCND1
Luteolin	BCL2L1
Luteolin	CDKN1A
Luteolin	CASP9
Luteolin	MMP2
Luteolin	MMP9
Luteolin	MAPK1
Luteolin	IL10
Luteolin	RB1
Luteolin	CDK4
Luteolin	TNF
Luteolin	JUN
Luteolin	IL6
Luteolin	CASP3
Luteolin	TP53
Luteolin	NFKBIA
Luteolin	XDH

Luteolin	TOP1
Luteolin	MDM2
Luteolin	APP
Luteolin	MMP1
Luteolin	PCNA
Luteolin	ERBB2
Luteolin	PPARG
Luteolin	HMOX1
Luteolin	CASP7
Luteolin	ICAM1
Luteolin	MCL1
Luteolin	BIRC5
Luteolin	IL2
Luteolin	CCNB1
Luteolin	TYR
Luteolin	IFNG
Luteolin	IL4
Luteolin	TOP2A
Luteolin	GSTP1
Luteolin	XIAP
Luteolin	SLC2A4
Luteolin	INSR
Luteolin	CD40LG
Luteolin	NUF2
Luteolin	ADCY2
Luteolin	MET
Magnograndiolide	GABRA2
Magnograndiolide	GABRA1
Magnograndiolide	GRIA2
Magnograndiolide	GABRA6
Mandenol	PTGS1
Mandenol	PTGS2
Mandenol	NCOA2
MOL004388	PTGS1
MOL004388	F10
MOL004388	PTGS2
MOL004388	TOP2A
MOL004388	KCNMA1
MOL004396	ESR1
MOL004396	SCN5A
MOL004396	PTGS2
MOL004396	ADRA1B
MOL004396	ADRB2
MOL004396	CDK2
MOL004396	PIM1
MOL004396	CCNA2
MOL004396	NCOA2
MOL004396	CALM1

MOL004396	CALM2
MOL004396	CALM3
MOL006957	AR
MOL006957	PTGS2
MOL006957	ADRB2
MOL006957	CALM1
MOL006957	CALM2
MOL006957	CALM3
MOL009495	PTGS1
MOL009495	KCNH2
MOL009495	SCN5A
MOL009495	F10
MOL009495	PTGS2
MOL009495	F7
MOL009495	RXRA
MOL009495	TOP2A
MOL009495	PIK3CG
MOL009495	NCOA2
MOL009495	NCOA1
MOL009495	KCNMA1
MOL009495	CALM1
MOL009495	CALM2
MOL009495	CALM3
MOL009496	PTGS1
MOL009496	F10
MOL009496	PTGS2
MOL009496	F7
MOL009496	TOP2A
MOL009496	IGHG1
MOL009496	NCOA2
MOL009496	NCOA1
MOL009496	CALM1
MOL009496	CALM2
MOL009496	CALM3
MOL009500	PTGS1
MOL009500	SCN5A
MOL009500	F10
MOL009500	PTGS2
MOL009500	F7
MOL009500	RXRA
MOL009500	TOP2A
MOL009500	PIK3CG
MOL009500	NCOA2
MOL009500	NCOA1
MOL009500	CALM1
MOL009500	CALM2
MOL009500	CALM3
MOL009504	PTGS1

MOL009504	PTGS2
MOL009504	PDE3A
MOL009504	ADRB2
MOL009504	GABRA1
MOL009504	PIK3CG
MOL009504	CHRNA7
MOL009504	IGHG1
MOL009504	NCOA2
MOL009504	PKIA
MOL009513	PTGS1
MOL009513	SCN5A
MOL009513	F10
MOL009513	PTGS2
MOL009513	F7
MOL009513	RXRA
MOL009513	PIK3CG
MOL009513	NCOA2
MOL009513	KCNMA1
MOL009513	CALM1
MOL009513	CALM2
MOL009513	CALM3
MOL009519	PTGS1
MOL009519	SCN5A
MOL009519	F10
MOL009519	PTGS2
MOL009519	TOP2A
MOL009519	PIK3CG
MOL009519	NCOA2
MOL009519	CALM1
MOL009519	CALM2
MOL009519	CALM3
Ohioensin A	PTGS1
Ohioensin A	PTGS2
Ohioensin A	TOP2A
Olivil	PTGS2
Olivil	NCOA2
Olivil	CALM1
Olivil	CALM2
Olivil	CALM3
Panaxadiol	NR3C1
Poriferast-5-en-3b	PGR
Poriferast-5-en-3b	NCOA2
Quercetin	PTGS1
Quercetin	AR
Quercetin	PPARG
Quercetin	PTGS2
Quercetin	HSP90AA1
Quercetin	PIK3CG

Quercetin	NCOA2
Quercetin	DPP4
Quercetin	AKR1B1
Quercetin	PRSS1
Quercetin	TOP2A
Quercetin	F2
Quercetin	KCNH2
Quercetin	SCN5A
Quercetin	F10
Quercetin	ADRB2
Quercetin	MMP3
Quercetin	F7
Quercetin	NOS3
Quercetin	RXRA
Quercetin	ACHE
Quercetin	GABRA1
Quercetin	MAOB
Quercetin	RELA
Quercetin	EGFR
Quercetin	AKT1
Quercetin	VEGFA
Quercetin	CCND1
Quercetin	BCL2
Quercetin	BCL2L1
Quercetin	FOS
Quercetin	CDKN1A
Quercetin	EIF6
Quercetin	BAX
Quercetin	CASP9
Quercetin	PLAU
Quercetin	MMP2
Quercetin	MAPK1
Quercetin	IL10
Quercetin	EGF
Quercetin	RB1
Quercetin	TNF
Quercetin	JUN
Quercetin	IL6
Quercetin	AHSA1
Quercetin	CASP3
Quercetin	TP53
Quercetin	ELK1
Quercetin	NFKBIA
Quercetin	POR
Quercetin	ODC1
Quercetin	XDH
Quercetin	CASP8
Quercetin	TOP1

Quercetin	RAF1
Quercetin	SOD1
Quercetin	PRKCA
Quercetin	MMP1
Quercetin	HIF1A
Quercetin	STAT1
Quercetin	RUNX1T1
Quercetin	CDK1
Quercetin	HSPA5
Quercetin	ERBB2
Quercetin	ACACA
Quercetin	HMOX1
Quercetin	CYP3A4
Quercetin	CYP1A2
Quercetin	CAV1
Quercetin	MYC
Quercetin	F3
Quercetin	GJA1
Quercetin	CYP1A1
Quercetin	ICAM1
Quercetin	IL1B
Quercetin	CCL2
Quercetin	SELE
Quercetin	VCAM1
Quercetin	PTGER3
Quercetin	CXCL8
Quercetin	PRKCB
Quercetin	BIRC5
Quercetin	DUOX2
Quercetin	HSPB1
Quercetin	TGFB1
Quercetin	SULT1E1
Quercetin	MGAM
Quercetin	IL2
Quercetin	NR1I2
Quercetin	CYP1B1
Quercetin	CCNB1
Quercetin	PLAT
Quercetin	THBD
Quercetin	SERPINE1
Quercetin	COL1A1
Quercetin	IFNG
Quercetin	ALOX5
Quercetin	PTEN
Quercetin	IL1A
Quercetin	MPO
Quercetin	NCF1
Quercetin	ABCG2

Quercetin	HAS2
Quercetin	GSTP1
Quercetin	NFE2L2
Quercetin	NQO1
Quercetin	PARP1
Quercetin	AHR
Quercetin	PSMD3
Quercetin	SLC2A4
Quercetin	COL3A1
Quercetin	CXCL11
Quercetin	CXCL2
Quercetin	DCAF5
Quercetin	NR1I3
Quercetin	CHEK2
Quercetin	INSR
Quercetin	CLDN4
Quercetin	PPARA
Quercetin	PPARD
Quercetin	HSF1
Quercetin	CRP
Quercetin	CXCL10
Quercetin	CHUK
Quercetin	SPP1
Quercetin	RUNX2
Quercetin	RASSF1
Quercetin	E2F1
Quercetin	E2F2
Quercetin	ACPP
Quercetin	CTSD
Quercetin	IGFBP3
Quercetin	IGF2
Quercetin	CD40LG
Quercetin	IRF1
Quercetin	ERBB3
Quercetin	PON1
Quercetin	DIO1
Quercetin	PCOLCE
Quercetin	NPEPPS
Quercetin	HK2
Quercetin	NKX3-1
Quercetin	RASA1
Quercetin	GSTM1
Quercetin	GSTM2
Rhein	PTGS1
Rhein	PTGS2
Rhein	PIK3CG
Rhein	NCOA2
Rhein	AKR1B1

Rhein	JUN
Sitosterol	PGR
Sitosterol	NCOA2
Sitosterol	NR3C2
Stigmasterol	PGR
Stigmasterol	NR3C2
Stigmasterol	NCOA2
Stigmasterol	ADH1C
Stigmasterol	IGHG1
Stigmasterol	RXRA
Stigmasterol	NCOA1
Stigmasterol	PTGS1
Stigmasterol	PTGS2
Stigmasterol	ADRA2A
Stigmasterol	SLC6A2
Stigmasterol	SLC6A3
Stigmasterol	ADRB2
Stigmasterol	AKR1B1
Stigmasterol	PLAU
Stigmasterol	LTA4H
Stigmasterol	MAOB
Stigmasterol	MAOA
Stigmasterol	CTRB1
Stigmasterol	CHRM3
Stigmasterol	CHRM1
Stigmasterol	ADRB1
Stigmasterol	SCN5A
Stigmasterol	HTR2A
Stigmasterol	ADRA1A
Stigmasterol	GABRA3
Stigmasterol	CHRM2
Stigmasterol	ADRA1B
Stigmasterol	GABRA1
Stigmasterol	CHRNA7
Suchilactone	KCNH2
Suchilactone	SCN5A
Suchilactone	F10
Suchilactone	PTGS2
Suchilactone	F7
Suchilactone	ADRB2
Suchilactone	NCOA1
Suchilactone	KCNMA1
Suchilactone	PTGS1
Suchilactone	RXRA
Suchilactone	PDE3A
Suchilactone	ADRA1D
Suchilactone	CALM1
Suchilactone	CALM2

Suchilactone	CALM3
Taxifolin	PTGS1
Taxifolin	PTGS2
Taxifolin	PIK3CG
Taxifolin	RXRA
Taxifolin	AKR1B1
Taxifolin	RELA
Taxifolin	ICAM1
Taxifolin	DGAT2
Taxifolin	MTTP
Taxifolin	APOB
Toralactone	NOS2
Toralactone	PTGS1
Toralactone	ESR1
Toralactone	PTGS2
Toralactone	ESR2
Toralactone	PIK3CG
Toralactone	CHEK1
Yinyanghuo A	ESR1
Yinyanghuo A	AR
Yinyanghuo A	F10
Yinyanghuo A	PTGS2
Yinyanghuo A	TOP2A
Yinyanghuo A	PRSS1
Yinyanghuo A	PIM1
Yinyanghuo A	NCOA2
Yinyanghuo A	CALM1
Yinyanghuo A	CALM2
Yinyanghuo A	CALM3
Yinyanghuo C	NOS2
Yinyanghuo C	AR
Yinyanghuo C	F10
Yinyanghuo C	PTGS2
Yinyanghuo C	ACHE
Yinyanghuo C	ESR2
Yinyanghuo C	DPP4
Yinyanghuo C	CDK2
Yinyanghuo C	PRSS1
Yinyanghuo C	NCOA2
Yinyanghuo C	CALM1
Yinyanghuo C	CALM2
Yinyanghuo C	CALM3
Yinyanghuo E	NOS2
Yinyanghuo E	AR
Yinyanghuo E	F10
Yinyanghuo E	PTGS2
Yinyanghuo E	ACHE
Yinyanghuo E	TOP2A

Yinyanghuo E	DPP4
Yinyanghuo E	PRSS1
Yinyanghuo E	NCOA2
Yinyanghuo E	CALM1
Yinyanghuo E	CALM2
Yinyanghuo E	CALM3
