# Supplementary material for: Systems Biology and Chemoinformatics-Based Strategies to Explore the Biological Mechanism of Fugui Wenyang Decoction in Treating Vascular Dementia Rats
Source: Oxid Med Cell Longev. 2021 Oct 7;2021:6693955. doi: 10.1155/2021/6693955 (PMC8517630; doi:10.1155/2021/6693955)
Supplement: Supplementary 2 — Table S2: VD genes. [file 6693955.f2.pdf]

Table S2 VD Gene

MAPT
APP
APOE
PSEN1
ACE
TNF
ACHE
BCHE
SORL1
RETREG1
MTHFR
CHAT
NOTCH3
VEGFA
PLAU
NOS3
BLMH
SERPINA3
MPO
A2M
HFE
APBB2
PAXIP1
MT-ND1
MIR34A
MIR146A
MIR106B
MIR29A
MIR107
MIR29B1
MIR328
MIR298
AD5
AD10
AD6
AD7
AD11
AD12
AD13
AD14
AD15
AD16
AD17
AD8
PON1
CST3
PRNP

CRH
MBP
SOD1
IL1A
AGER
GFAP
SLC6A4
CTSD
GRIN1
GRIN2A
CAT
CASP3
GRIN2B
CHRNA4
COX5A
SNCA
BDNF
ITM2B
NGF
PSEN2
GRN
SYP
LTA
SQSTM1
TARDBP
BACE1
SNCB
IL1B
MAOB
IDE
CYP46A1
HMOX1
LRP1
S100B
CHRNA7
SNCG
HTR2A
ADAM17
TNFRSF1A
TF
CD40
VLDLR
CLU
DNM1L
GAL
VIP
REG1A
MT3

MIR210
NFE2L2
KEAP1
NQO1
HMOX1
SOD1
TFRC
IREB2
SLC40A1
GPX4
ATF4
CHOP
TRIB3
CHAC1
TP53
DDIT3
TFAP2C
SP1
NFE2L2
GSH
DPP4
ACSL4
AKR1C1
AKR1C2
AKR1C3
ALOX15
ATP5G3
CARS
CBS
CD44v
CHAC1
CISD1
CS
FANCD2
GCLM
GCLC
GLS2
GPX4
GSS
HMGCR
KOD
LPCAT3
MT1G
PTGS2
NCOA4
HSPB1
HSPB2
HSPB3

HSPB4
HSPB5
