# Supplementary material for: Systems Biology and Chemoinformatics-Based Strategies to Explore the Biological Mechanism of Fugui Wenyang Decoction in Treating Vascular Dementia Rats
Source: Oxid Med Cell Longev. 2021 Oct 7;2021:6693955. doi: 10.1155/2021/6693955 (PMC8517630; doi:10.1155/2021/6693955)
Supplement: Supplementary 3 — Table S3: biological processes of the FGWYD-VD PPI network. [file 6693955.f3.pdf]

Table S3 Biological Processes of FGWYD-VD PPI Network

Cluster	Term	Pathway	Count	%	Pvalue
	GO:0043066	negative regulation of apoptotic proce	21	36.84211	9.18E-18
	GO:0006954	inflammatory response	17	29.82456	6.06E-14
	GO:0007568	aging	12	21.05263	5.99E-12
	GO:0001666	response to hypoxia	11	19.29825	2.32E-10
	GO:0051092	positive regulation of NF-kappaB tran	10	17.54386	5.16E-10
	GO:0071456	cellular response to hypoxia	9	15.78947	9.62E-10
	GO:0001525	angiogenesis	11	19.29825	2.91E-09
	GO:0008284	positive regulation of cell proliferatio	14	24.5614	3.12E-09
	GO:0043065	positive regulation of apoptotic proces	10	17.54386	5.97E-07
	GO:0034097	response to cytokine	6	10.52632	7.93E-07
	GO:0043524	negative regulation of neuron apoptoti	7	12.2807	4.95E-06
	GO:0042542	response to hydrogen peroxide	5	8.77193	2.47E-05
	GO:0006979	response to oxidative stress	6	10.52632	3.22E-05
	GO:0043536	positive regulation of blood vessel en	4	7.017544	3.28E-05
	GO:0042127	regulation of cell proliferation	7	12.2807	3.39E-05
	GO:0045766	positive regulation of angiogenesis	6	10.52632	3.99E-05
	GO:0050679	positive regulation of epithelial cell p	5	8.77193	4.71E-05
	GO:0035924	cellular response to vascular endothel	4	7.017544	5.93E-05
	GO:0071677	positive regulation of mononuclear ce	3	5.263158	6.53E-05
	GO:0001938	positive regulation of endothelial cell	5	8.77193	8.16E-05
	GO:0050729	positive regulation of inflammatory re	5	8.77193	1.02E-04
	GO:2000352	negative regulation of endothelial cell	4	7.017544	1.08E-04
	GO:0045907	positive regulation of vasoconstrictior	4	7.017544	1.63E-04
	GO:0006919	activation of cysteine-type endopeptid	5	8.77193	1.68E-04
	GO:0043123	positive regulation of I-kappaB kinase	6	10.52632	1.96E-04
	GO:0032930	positive regulation of superoxide anio	3	5.263158	3.87E-04
	GO:0043525	positive regulation of neuron apoptoti	4	7.017544	3.94E-04
	GO:0030168	platelet activation	5	8.77193	5.83E-04
	GO:0090026	positive regulation of monocyte chem	3	5.263158	0.001272
**1**	GO:0030949	positive regulation of vascular endoth	3	5.263158	0.001272
	GO:0043066	negative regulation of apoptotic proce	11	23.91304	2.64E-07
	GO:0045766	positive regulation of angiogenesis	6	13.04348	1.36E-05
	GO:0006986	response to unfolded protein	4	8.695652	1.92E-04
	GO:0050999	regulation of nitric-oxide synthase act	3	6.521739	0.002191
	GO:0006366	transcription from RNA polymerase I	7	15.21739	0.002339
	GO:0006954	inflammatory response	6	13.04348	0.00332
	GO:0051402	neuron apoptotic process	3	6.521739	0.003517
	GO:0030968	endoplasmic reticulum unfolded prote	3	6.521739	0.006461
	GO:0001666	response to hypoxia	4	8.695652	0.010936
	GO:0050727	regulation of inflammatory response	3	6.521739	0.012364
	GO:0030512	negative regulation of transforming g	3	6.521739	0.012741
	GO:0038033	positive regulation of endothelial cell	2	4.347826	0.013329
	GO:2001028	positive regulation of endothelial cell	2	4.347826	0.013329

	GO:0045454	cell redox homeostasis	3	6.521739	0.018091
	GO:0071639	positive regulation of monocyte chem	2	4.347826	0.018612
	GO:1903071	positive regulation of ER-associated u	2	4.347826	0.021243
	GO:0071456	cellular response to hypoxia	3	6.521739	0.027306
	GO:1902176	negative regulation of oxidative stress	2	4.347826	0.036885
	GO:0043536	positive regulation of blood vessel en	2	4.347826	0.049734
	GO:2000352	negative regulation of endothelial cell	2	4.347826	0.072439
	GO:0001937	negative regulation of endothelial cell	2	4.347826	0.074929
**2**	GO:0043542	endothelial cell migration	2	4.347826	0.074929
	GO:0006940	regulation of smooth muscle contracti	4	25	3.21E-07
	GO:0071880	adenylate cyclase-activating adrenergi	4	25	5.54E-07
	GO:0071883	activation of MAPK activity by adren	3	18.75	4.46E-06
	GO:0032811	negative regulation of epinephrine sec	3	18.75	4.46E-06
	GO:0035625	epidermal growth factor-activated rec	3	18.75	4.46E-06
	GO:0007186	G-protein coupled receptor signaling p	8	50	5.43E-06
	GO:0007187	G-protein coupled receptor signaling p	4	25	8.55E-06
	GO:0010700	negative regulation of norepinephrine	3	18.75	1.56E-05
	GO:0007200	phospholipase C-activating G-protein	4	25	2.55E-05
	GO:0019229	regulation of vasoconstriction	3	18.75	1.40E-04
	GO:0007268	chemical synaptic transmission	4	25	0.001155
	GO:0007198	adenylate cyclase-inhibiting serotonin	2	12.5	0.002678
	GO:0007218	neuropeptide signaling pathway	3	18.75	0.003574
	GO:0030168	platelet activation	3	18.75	0.004606
	GO:0051967	negative regulation of synaptic transm	2	12.5	0.008013
	GO:0050795	regulation of behavior	2	12.5	0.008013
	GO:0007399	nervous system development	3	18.75	0.026397
**3**	GO:0007271	synaptic transmission, cholinergic	2	12.5	0.03256
	GO:0001994	norepinephrine-epinephrine vasocons	3	14.28571	8.07E-06
	GO:0007271	synaptic transmission, cholinergic	4	19.04762	1.09E-05
	GO:0008283	cell proliferation	6	28.57143	5.67E-05
	GO:0060079	excitatory postsynaptic potential	3	14.28571	5.00E-04
	GO:0042135	neurotransmitter catabolic process	2	9.52381	0.009491
	GO:0050804	modulation of synaptic transmission	2	9.52381	0.020065
	GO:0048169	regulation of long-term neuronal syna	2	9.52381	0.021234
	GO:0055114	oxidation-reduction process	4	19.04762	0.031803
**4**	GO:0007268	chemical synaptic transmission	3	14.28571	0.032636
	GO:0006750	glutathione biosynthetic process	3	37.5	1.78E-05
	GO:1901687	glutathione derivative biosynthetic pr	3	37.5	3.43E-05
	GO:2001237	negative regulation of extrinsic apopt	3	37.5	1.04E-04
	GO:0006749	glutathione metabolic process	3	37.5	2.27E-04
	GO:0097069	cellular response to thyroxine stimulu	2	25	8.34E-04
	GO:0006979	response to oxidative stress	3	37.5	8.74E-04
	GO:0051409	response to nitrosative stress	2	25	0.001667

	GO:0018916	nitrobenzene metabolic process	2	25	0.001667
	GO:0051900	regulation of mitochondrial depolariza	2	25	0.001667
	GO:0007568	aging	3	37.5	0.001951
**5**	GO:0008152	metabolic process	3	37.5	0.002022
	GO:1902476	chloride transmembrane transport	4	50	5.66E-06
	GO:0007214	gamma-aminobutyric acid signaling pa	3	37.5	3.43E-05
	GO:0034220	ion transmembrane transport	4	50	6.50E-05
	GO:0007165	signal transduction	4	50	0.009342
	GO:0006821	chloride transport	2	25	0.016559
	GO:0043524	negative regulation of neuron apoptoti	2	25	0.053755
**6**	GO:0007268	chemical synaptic transmission	2	25	0.095875
	GO:0007268	chemical synaptic transmission	5	55.55556	2.72E-06
	GO:0015844	monoamine transport	3	33.33333	5.55E-06
	GO:0042493	response to drug	5	55.55556	6.96E-06
	GO:0045471	response to ethanol	3	33.33333	0.001058
	GO:0051583	dopamine uptake involved in synaptic	2	22.22222	0.00238
	GO:0055085	transmembrane transport	3	33.33333	0.005558
	GO:0042136	neurotransmitter biosynthetic process	2	22.22222	0.005704
	GO:0048265	response to pain	2	22.22222	0.008072
	GO:0051412	response to corticosterone	2	22.22222	0.008545
	GO:0001975	response to amphetamine	2	22.22222	0.014677
	GO:0007611	learning or memory	2	22.22222	0.017026
	GO:0035094	response to nicotine	2	22.22222	0.017496
	GO:0060291	long-term synaptic potentiation	2	22.22222	0.017965
	GO:0019233	sensory perception of pain	2	22.22222	0.024512
	GO:0007613	memory	2	22.22222	0.029165
	GO:0007626	locomotory behavior	2	22.22222	0.039333
**7**	GO:0001666	response to hypoxia	2	22.22222	0.079081
	GO:0007190	activation of adenylate cyclase activity	4	50	4.35E-07
	GO:0007189	adenylate cyclase-activating G-protein	3	37.5	1.81E-04
	GO:0046960	sensitization	2	25	8.34E-04
	GO:0002025	vasodilation by norepinephrine-epinep	2	25	0.00125
	GO:0031649	heat generation	2	25	0.00125
	GO:0071870	cellular response to catecholamine sti	2	25	0.002915
	GO:0002024	diet induced thermogenesis	2	25	0.003746
	GO:0010579	positive regulation of adenylate cyclas	2	25	0.003746
	GO:0060158	phospholipase C-activating dopamine	2	25	0.004162
	GO:0040015	negative regulation of multicellular or	2	25	0.004993
	GO:0007191	adenylate cyclase-activating dopamine	2	25	0.004993
	GO:0001963	synaptic transmission, dopaminergic	2	25	0.005408
	GO:0060292	long term synaptic depression	2	25	0.006652
	GO:0001659	temperature homeostasis	2	25	0.007895
	GO:0071880	adenylate cyclase-activating adrenergi	2	25	0.007895

	GO:0019226	transmission of nerve impulse	2	25	0.008309
	GO:0046627	negative regulation of insulin receptor	2	25	0.012029
	GO:0001975	response to amphetamine	2	25	0.012854
	GO:0050873	brown fat cell differentiation	2	25	0.013266
	GO:0009409	response to cold	2	25	0.014914
	GO:0030819	positive regulation of cAMP biosynthe	2	25	0.016559
	GO:0007612	learning	2	25	0.023525
	GO:0007416	synapse assembly	2	25	0.025158
	GO:0006874	cellular calcium ion homeostasis	2	25	0.038137
	GO:0030168	platelet activation	2	25	0.046974
	GO:0018105	peptidyl-serine phosphorylation	2	25	0.050968
**8**	GO:0007268	chemical synaptic transmission	2	25	0.095875
	GO:0032469	endoplasmic reticulum calcium ion ho	2	25	0.006237
	GO:2001234	negative regulation of apoptotic signa	2	25	0.006652
	GO:0006509	membrane protein ectodomain proteol	2	25	0.009137
	GO:0051402	neuron apoptotic process	2	25	0.013678
	GO:0043066	negative regulation of apoptotic proce	3	37.5	0.014056
**9**	GO:0001764	neuron migration	2	25	0.042966
	GO:0008202	steroid metabolic process	4	80	6.25E-08
	GO:0042574	retinal metabolic process	3	60	2.81E-06
	GO:0008210	estrogen metabolic process	2	40	0.002618
	GO:0055114	oxidation-reduction process	3	60	0.007101
	GO:0042572	retinol metabolic process	2	40	0.007128
**10**	GO:0001523	retinoid metabolic process	2	40	0.014453
	GO:0007596	blood coagulation	3	100	1.19E-04
	GO:0010544	negative regulation of platelet activati	2	66.66667	9.53E-04
	GO:0051918	negative regulation of fibrinolysis	2	66.66667	0.001191
**11**	GO:0042730	fibrinolysis	2	66.66667	0.0025
	GO:0007598	blood coagulation, extrinsic pathway	2	66.66667	5.95E-04
	GO:0017187	peptidyl-glutamic acid carboxylation	2	66.66667	0.00131
	GO:0006465	signal peptide processing	2	66.66667	0.002975
	GO:0051897	positive regulation of protein kinase E	2	66.66667	0.00998
	GO:0006888	ER to Golgi vesicle-mediated transpor	2	66.66667	0.018966
	GO:0007596	blood coagulation	2	66.66667	0.021796
**12**	GO:0030335	positive regulation of cell migration	2	66.66667	0.021796
	GO:0019430	removal of superoxide radicals	2	33.33333	0.003568
	GO:0060291	long-term synaptic potentiation	2	33.33333	0.011265
	GO:0009636	response to toxic substance	2	33.33333	0.025058
	GO:0045471	response to ethanol	2	33.33333	0.03088
	GO:0007417	central nervous system development	2	33.33333	0.035228
	GO:0007605	sensory perception of sound	2	33.33333	0.038984

	GO:0007568	aging	2	33.33333	0.04818
**13**	GO:0043065	positive regulation of apoptotic proces	2	33.33333	0.086203

Genes	Fold Enrichment	Bonferroni
IL10, CDKN1A, NGF, MPO, MMP9, EGFR, IL2, RI	13.59676113	1.39E-14
IL10, CRP, CD40, TGFB1, CXCL8, FOS, PTGS2, T	13.21409064	9.21E-11
IL10, CASP9, IL6, JUN, TGFB1, VCAM1, CAT, CC	21.42519936	9.10E-09
TGFB1, VCAM1, NOS2, PLAU, CASP3, MMP2, CA	18.84047328	3.52E-07
AR, IL6, CD40, TGFB1, CD40LG, IL1B, CAT, TNF	22.15011212	7.84E-07
CCNB1, MDM2, HMOX1, AKT1, PTGS2, HIF1A, T	27.61842105	1.46E-06
JUN, CXCL8, EGF, MMP2, SERPINE1, HMOX1, C	14.53166549	4.42E-06
TGFB1, EGF, EGFR, IL2, RELA, VEGFA, CXCL10	8.850538363	4.74E-06
IL6, MAPK8, TGFB1, HMOX1, AKT1, NGF, PTGS	9.819883041	9.06E-04
IL4, JUN, STAT1, FOS, BCL2L1, MCL1	33.99190283	0.001203234
JUN, BDNF, HMOX1, CCL2, APOE, NGF, BCL2L1	15.6225412	0.007493541
JUN, STAT1, CASP3, CAT, HMOX1	28.88200894	0.036779447
HMOX1, AKT1, APOE, PTGS2, MPO, EGFR	16.06889952	0.04777968
TGFB1, AKT1, MAPK14, VEGFA	62.02031394	0.048571195
CXCL10, JUN, CD40, NOS2, PLAU, TNF, TNFRSF	11.14689426	0.05017187
CXCL8, IL1B, SERPINE1, HMOX1, HIF1A, VEGF	15.37025172	0.058821308
IL6, TGFB1, MYC, EGFR, VEGFA	24.5497076	0.069015142
VCAM1, AKT1, MAPK14, VEGFA	51.23417239	0.086200355
IL4, TGFB1, TNF	220.9473684	0.094380335
JUN, CCL2, AKT1, HIF1A, VEGFA	21.34757183	0.116613151
SERPINE1, CCL2, EGFR, IL2, TNFRSF1A	20.17784186	0.143220834
IL10, IL4, SERPINE1, ICAM1	42.08521303	0.151930483
AKT1, PTGS2, EGFR, ICAM1	36.8245614	0.218985901
CASP8, CASP3, PPARG, NGF, TNF	17.74677658	0.22478281
CD40, CASP8, HMOX1, TNF, RELA, TNFRSF1A	10.97875123	0.257522196
CRP, TGFB1, EGFR	98.19883041	0.444877291
CASP9, JUN, CASP3, TP53	27.40432477	0.450785754
IL6, CD40, CD40LG, MAPK1, AKT1	12.8085431	0.587425322
CXCL10, SERPINE1, CCL2	55.23684211	0.855366298
IL1B, HIF1A, VEGFA	55.23684211	0.855366298
NFKBIA, IKBKB, GSK3B, HSPA5, PTEN, CDK1, I	8.825226947	2.52E-04
IL1A, NOS3, KDR, HSPB1, F3, NFE2L2	19.04574669	0.012873202
HSP90AA1, HSPB2, HSPB1, HSPB3	34.76604555	0.167630009
HSP90AA1, NOS3, CAV1	42.12040134	0.877133439
PARP1, IRF1, AHR, NR3C1, ESR1, RUNX2, NFE2	4.981100093	0.893367212
IKBKB, IL1A, SELE, AGER, CXCL2, NFE2L2	5.779052426	0.958396169
RB1, APP, CASP7	33.18577075	0.965547778
CCND1, HSPA5, NFE2L2	24.33623188	0.997964688
ADAM17, CAV1, RAF1, AGER	8.489383215	0.999972791
XIAP, SELE, ESR1	17.38302277	0.999993165
ADAM17, HSPA5, CAV1	17.11141304	0.999995256
KDR, HSPB1	146.0173913	0.999997317
HSPB1, MET	146.0173913	0.999997317

NCF1, NOS3, NFE2L2	14.22247318	0.999999974
IL1A, AGER	104.2981366	0.999999984
CAV1, NFE2L2	91.26086957	0.999999999
CCNA2, PTEN, NFE2L2	11.4076087	1
HSPB1, NFE2L2	52.14906832	1
HSPB1, NFE2L2	38.42562929	1
KDR, NFE2L2	26.07453416	1
GJA1, CAV1	25.17541229	1
NOS3, PTEN	25.17541229	1
CHRM2, ADRA2C, ADRA2B, ADRA2A	262.375	9.17E-05
DRD2, ADRA2C, ADRA2B, ADRA2A	220.9473684	1.58E-04
ADRA2C, ADRA2B, ADRA2A	787.125	0.001275948
ADRA2C, ADRA2B, ADRA2A	787.125	0.001275948
ADRA2C, ADRA2B, ADRA2A	787.125	0.001275948
CHRM2, OPRD1, CXCL11, PTGER3, HTR1A, ADI	9.339265851	0.001550799
CHRM2, OPRD1, HTR1B, OPRM1	91.26086957	0.002443574
ADRA2C, ADRA2B, ADRA2A	449.7857143	0.004451839
OPRD1, PTGER3, HTR1B, OPRM1	63.60606061	0.007269939
ADRA2C, ADRA2B, ADRA2A	157.425	0.039307568
OPRD1, HTR1A, HTR1B, OPRM1	17.49166667	0.281517306
HTR1A, HTR1B	699.6666667	0.535514349
OPRD1, GAL, OPRM1	31.17326733	0.640843074
ADRA2C, ADRA2B, ADRA2A	27.37826087	0.732944289
HTR1B, DRD2	233.2222222	0.899829862
HTR1A, HTR1B	233.2222222	0.899829862
CHRM2, GAL, APOB	10.97038328	0.999524353
CHRM2, CHRM4	56.72972973	0.999922644
ADRA1D, ADRA1B, ADRA1A	599.7142857	0.002918842
CHRM3, CHRM1, CHRM5, HTR3A	86.44530245	0.003952586
CHRM3, CHRM1, CHRM5, CYP1A1, ADRA1D, A	13.10850898	0.020302666
PPP3CA, GRIN1, SNCA	85.67346939	0.165622518
MAOB, MAOA	199.9047619	0.968318973
PPP3CA, HRH1	94.07282913	0.999349356
GRIN1, SNCA	88.84656085	0.999577533
MAOB, MAOA, CYP1A1, SNCA	5.402831403	0.999991703
HTR2C, HTR2A, GRIN1	9.995238095	0.999993925
GCLC, GSS, GCLM	393.5625	0.002296881
GSTM2, GSTM1, GSTP1	286.2272727	0.004411586
GCLC, GSTP1, GCLM	165.7105263	0.013323649
GSTM2, GSTM1, GSTP1	112.4464286	0.028855502
GCLC, GCLM	2099	0.101992485
GCLC, GSS, GCLM	57.24545455	0.106672395
GCLC, GCLM	1049.5	0.193592839

GSTM2, GSTM1	1049.5	0.193592839
GCLC, GCLM	1049.5	0.193592839
GCLC, GSS, GCLM	38.16363636	0.222711715
GSTM2, GSTM1, GSTP1	37.48214286	0.229773369
GABRA6, GABRA3, GABRE, GABRG3	90.27956989	3.96E-04
GABRA6, GABRA3, GABRG3	286.2272727	0.002396304
GRIA2, GABRA6, GABRA3, GABRG3	39.98095238	0.004542204
GRIA2, CLDN4, GABRA6, GABRE	7.231696813	0.481613899
GABRA6, GABRG3	104.95	0.689267851
SNCB, MT3	31.8030303	0.979094588
GRIA2, SNCB	17.49166667	0.999137033
GRIN2A, CHRNA4, CRH, SLC6A2, SLC6A4	38.87037037	4.87E-04
SLC6A2, SLC6A3, SLC6A4	699.6666667	9.94E-04
GRIN2A, CRH, SLC6A2, SLC6A3, SLC6A4	30.6871345	0.001245444
GRIN2A, CRH, SLC6A3	53.30793651	0.172630596
SLC6A2, SLC6A3	746.3111111	0.347240124
SLC6A2, SLC6A3, SLC6A4	22.93989071	0.631233037
CHAT, SLC6A3	310.962963	0.640817187
CRH, SLC6A2	219.503268	0.765611406
CRH, CALM1	207.308642	0.784794172
GRIN2A, CALM1	120.3727599	0.929110665
GRIN2A, CRH	103.654321	0.953762791
CHRNA4, SLC6A3	100.8528529	0.957551035
GRIN2A, CRH	98.19883041	0.961029104
GRIN2A, CHRNA4	71.76068376	0.988231166
GRIN2A, SLC6A4	60.18637993	0.994999269
CHRNA4, SLC6A3	44.42328042	0.99924053
CHRNA4, SLC6A4	21.69509044	0.999999606
ADRB1, DRD1, ADRB2, DRD5	209.9	8.10E-05
DRD1, ADRB2, DRD5	125.94	0.033062621
DRD1, DRD5	2099	0.143679704
ADRB1, ADRB2	1399.333333	0.207587448
ADRB1, ADRB2	1399.333333	0.207587448
DRD1, DRD5	599.7142857	0.418979157
ADRB1, ADRB2	466.4444444	0.502492252
DRD1, DRD5	466.4444444	0.502492252
DRD1, DRD5	419.8	0.539636476
ADRB1, ADRB2	349.8333333	0.605817815
DRD1, DRD5	349.8333333	0.605817815
DRD1, DRD5	322.9230769	0.635252753
DRD1, DRD5	262.375	0.711021816
ADRB1, DRD1	220.9473684	0.771060883
ADRB1, ADRB2	220.9473684	0.771060883

DRD1, DRD5	209.9	0.788163416
PRKCB, PRKCD	144.7586207	0.894697399
DRD1, DRD5	135.4193548	0.909851446
ADRB1, ADRB2	131.1875	0.916590488
ADRB1, ADRB2	116.6111111	0.938874461
ADRB1, DRD1	104.95	0.955208296
DRD1, DRD5	73.64912281	0.988060645
ACHE, DRD1	68.81967213	0.991254467
PRKCB, DRD5	45.13978495	0.999277123
PRKCB, PRKCD	36.50434783	0.999870136
PRKCB, PRKCD	33.584	0.999940535
GRIN2B, DRD5	17.49166667	0.999999993
BCL2, PSEN1	279.8666667	0.78409962
BCL2, PSEN1	262.375	0.805083262
BACE1, PSEN1	190.8181818	0.894471953
BCL2, PSEN1	127.2121212	0.965757047
BCL2, PSEN1, SQSTM1	13.83956044	0.968828286
MAPT, PSEN1	39.98095238	0.999978756
SULT1E1, NR1I2, AKR1C3, CYP1B1	312.4093023	5.62E-06
AKR1C1, AKR1C3, CYP1B1	839.6	2.53E-04
SULT1E1, CYP1B1	610.6181818	0.210160045
AKR1C1, AKR1C3, CYP1B1	17.01891892	0.473431333
AKR1C3, CYP1B1	223.8933333	0.474705482
AKR1C1, AKR1C3	110.1114754	0.730251162
THBD, PLAT, F2	91.26086957	0.005241166
THBD, F2	1399.333333	0.041068816
THBD, F2	1119.466667	0.05107269
PLAT, F2	533.0793651	0.104277443
F7, F10	2238.933333	0.017124888
F7, F10	1017.69697	0.037294631
F7, F10	447.7866667	0.082788841
F7, F10	133.2698413	0.25239085
F7, F10	69.96666667	0.426105943
F7, F10	60.84057971	0.472215655
F7, F10	60.84057971	0.472215655
NQO1, SOD1	466.4444444	0.337083232
S100B, GFAP	147.2982456	0.728242393
NQO1, MBP	65.85098039	0.945977373
NQO1, SOD1	53.30793651	0.972871797
MBP, S100B	46.64444444	0.983825919
MBP, SOD1	42.08521303	0.98967235

NQO1, SOD1	33.92323232	0.996581774
S100B, SOD1	18.65777778	0.999968542
