# Supplementary material for: Systems Biology and Chemoinformatics-Based Strategies to Explore the Biological Mechanism of Fugui Wenyang Decoction in Treating Vascular Dementia Rats
Source: Oxid Med Cell Longev. 2021 Oct 7;2021:6693955. doi: 10.1155/2021/6693955 (PMC8517630; doi:10.1155/2021/6693955)
Supplement: Supplementary 4 — Table S4: signaling pathway of the FGWYD-VD PPI network. [file 6693955.f4.pdf]

Table S4 Signaling Pathway of FGWYD-VD PPI Network

Term	Pathway	Count	%	Pvalue
hsa04668	TNF signaling pathway	29	8.978328	3.63E-16
hsa04066	HIF-1 signaling pathway	24	7.430341	1.49E-12
hsa04080	Neuroactive ligand-receptor interactio	40	12.3839	2.11E-12
hsa04151	PI3K-Akt signaling pathway	39	12.0743	6.28E-09
hsa04722	Neurotrophin signaling pathway	22	6.811146	7.07E-09
hsa04915	Estrogen signaling pathway	20	6.19195	7.77E-09
hsa04064	NF-kappa B signaling pathway	18	5.572755	3.70E-08
hsa04068	FoxO signaling pathway	22	6.811146	5.33E-08
hsa04726	Serotonergic synapse	20	6.19195	5.49E-08
hsa04370	VEGF signaling pathway	14	4.334365	5.41E-07
hsa04728	Dopaminergic synapse	20	6.19195	5.62E-07
hsa04071	Sphingolipid signaling pathway	19	5.882353	9.61E-07
hsa04725	Cholinergic synapse	18	5.572755	1.44E-06
hsa00480	Glutathione metabolism	8	2.47678	0.003718
hsa04724	Glutamatergic synapse	10	3.095975	0.036795

Genes	Fold Enrichment	Bonferroni
PTGS2, TNF, CXCL2, RELA, PIK3CG, ICAM1, IKBKB, CA	6.804386384	7.93E-14
EGLN1, CDKN1A, NOS2, TFRC, PRKCB, NOS3, EGF, INS	6.276459854	3.55E-10
CHRM2, OPRD1, GRIA2, CHRM3, PRSS1, CHRM1, CHRN	3.625391973	5.03E-10
CHRM2, GSK3B, CDKN1A, CHRM1, PTEN, RELA, EGFR,	2.838051412	1.49E-06
GSK3B, JUN, BDNF, PRKCD, PSEN2, PSEN1, NGF, MAPK	4.602737226	1.68E-06
JUN, HSP90AA1, NOS3, MMP2, PRKCD, ADCY2, FOS, OI	5.071886751	1.85E-06
CD40, VCAM1, CXCL8, CHUK, XIAP, PTGS2, TNF, RELA	5.194311603	8.80E-06
IL10, CDKN1A, TGFB1, CHUK, EGF, INSR, PTEN, SLC2A	4.121854232	1.27E-05
APP, MAOB, MAOA, PRKCB, ALOX15, HTR1A, HTR1B, [illegible]	4.52357467	1.31E-05
PRKCB, NOS3, HSPB1, PRKCA, PTGS2, MAPK14, PIK3C	5.761995932	1.29E-04
GRIA2, GSK3B, MAOB, MAOA, PRKCB, PRKCA, FOS, M	3.922787409	1.34E-04
OPRD1, PRKCB, NOS3, PRKCE, PTEN, PRKCA, MAPK14	3.975091241	2.29E-04
CHRM2, ACHE, CHRM3, CHRM1, PRKCB, CHRNA4, CHI	4.071217203	3.43E-04
GSTM2, GCLC, GSTM1, GPX4, GSTP1, GSS, ODC1, GCLI	3.938170889	0.587877883
PPP3CA, GRIA2, GRIN2A, PRKCB, GLS2, MAPK1, PRKC	2.202266615	0.999866644
