# Supplementary material for: Systems Biology and Chemoinformatics-Based Strategies to Explore the Biological Mechanism of Fugui Wenyang Decoction in Treating Vascular Dementia Rats
Source: Oxid Med Cell Longev. 2021 Oct 7;2021:6693955. doi: 10.1155/2021/6693955 (PMC8517630; doi:10.1155/2021/6693955)
Supplement: Supplementary 5 — Table S5: proteomics data and other rat proteins. [file 6693955.f5.pdf]

Table S5 Proteomics data and other rat proteins

Palb2
Brca2
Blm
Exo1
Rad51c
Topbp1
Bard1
Mus81
Brip1
Wrn
Tbc1d32
Fuz
Fam149b
Prickle1
Dzip1l
Gtl3
Uevld
Zw10
Stx18
Rint1
Sec22b
Nbas
Use1
Scfd2
Cog4
ENSMUSG00000020133
Acer1
Gtpbp8
Eri1
Sidt1
Nxph4
Rap1gds1
Igdcc3
Acer2
Ganc
Plrg1
Eftud2
Cdc5l
Prpf19
Cdc40
Aqr
Isy1
Syf2
Xab2
Prpf8
Rxfp1
Sspo

Hmcn1
Fras1
Lgr4
Nrg2
Frem1
Fn1
Grip1
Rxfp2
Tubb4b
Tubb4a
Tubal3
Tubb2a
Tubb3
Tubb2b
Gja1
Kcne1
Kcne3
Cdk5rap2
Akap9
Prkar2b
Clasp1
Cep135
Pcm1
Mief1
ENSMUSG00000039195
Rhot2
Fis1
Gdap1
Mrps33
ENSMUSG00000019797
Ccdc106
Emc2
Sh3glb1
Ptp4a2
Ptp4a3
Ptp4a1
Fam58b
Hpse2
Rprml
Allc
Cables2
Bcas3
Crabp2
Ndufa8
Ndufs7
Ndufv1
Ecsit
Tmem126b

Ndufaf1
Ndufaf4
Acad9
Ndufb7
Phlpp1
Phlpp2
Pten
Akt3
Akt2
Fkbp5
Cap1
Usp46
Calm1
Camk1
Calm2
Calml4
Calml3
Calm5
Calm4
Creb1
Nos3
Fbxl12
Tfb2m
Gm1818
Tefm
Utp6
Crlf3
Mterfd2
Adap2
Trmt10c
Trim36
Tmlhe
Ppia
Trat1
Lrch2
Aldh9a1
Ppp3r1
Ppp3cb
Nfatc1
Ppp3cc
Ppp3ca
Nfatc3
Nfatc2
Ppp3r2
Plcg2
103996542
103975182
103995055

103987855
103986698
GSMUA_Achr1P04850_001
103975738
103975098
103971246
Isca1
Tmem14c
Iba57
Nsmce4a
Tmem141
Slc22a4
Abcb10
Ankrd16
Psmb2
Psma1
Psmc2
Psma5
Psma4
Psmb4
Psmc5
Psmc3
Psma3
rCG_58522
Numa1
Gpr1
Tpk1
Gpr22
Zdbf2
Gpr3
Gpx2
Tceb1
Med1
Tsc22d4
Mlf1
Tceb2
Tsc22d3
Tsc22d2
Ptchd2
Fam117b
Tsc22d1
LOC685069
H3f3c
H2afx
H2afb3
Hist1h2bk
LOC690131
H3.3b

Hist1h2ba
Hist1h2bh
Rrp8
Sufu
Gli1
Ptch1
Gli3
Gli2
Ptch2
Stk36
Kif27
Spag16
Rpa2
Rpa3
Rad52
Atm
Top3a
Atr
Rad51d
Ypel4
C1ql3
Bai3
Fndc5
Pvalb
Tmem163
Neurod6
Gng2
Gnai1
Gnb1
Gnao1
Gnai2
Gnb4
Gnb2
Gnb5
Gnaq
Adrbk1
Prdm1
Bcl6
Bach2
Il2
Irf4
Maf
Rbm39
Slc24a5
Tpcn2
Slc24a4
Orai1
Hnrnpm

Fyn
Srsf1
Hnrnpa1
Hnrnpk
Khdrbs1
Prmt1
Khsrp
Xpo1
Ranbp1
Rcc1
Kpna4
Kpna3
Kpna7
Gm10184
Ppm1a
Kpna2
Nup214
Tbk1
Ikbke
Dhx8
Dhx9
Eif4e
D1Pas1
Eif2s3x
Dhx36
Ptpn11
Cd24a
Hmgb1
Cd72
Ddx58
Siglecg
Cbl
Il10
Kmt2a
Rnf14
Fancm
Fancd2
Faap100
Faap24
Fanca
Stra13
Fanci
Apitd1
Ercc4
Atat1
Nedd8
Bsdc1
Has3

Myh11
Myl9
Ppp1r12a
Myh10
Acta2
Actg2
Ttn
Rock2
Mylk
Acot11
Lix1
Pgm1
Fam26d
Rubcnl
Tmco5b
Mocs3
Cd300lg
Fbxo47
Krt90
Tpi1
Pgk1-rs7
Gapdhs
Bpgm
Pgk1
Aldoa
Aldoc
Aldoart1
Gpi1
Pgk2
Skp2
Rnf123
Ubc
Trip12
Cul1
Hecw2
Fbxw10
Ubac1
Rchy1
Mrpl16
Mrpl13
Mrps7
Mrpl43
Mrps12
Mrpl17
Mrps16
Mrps2
Mrpl12
Mrps11

Tmem108
Ndufa7
Zfand6
Cdv3
Qk1
Sugp1
Tsfm
Map2k6
Map3k5
Map3k6
Slc10a6
Olfr1143
Ube3c
Tmem106b
Gmppa
Fam96b
Mms19
Ciao1
Nubp1
Ndor1
Fam96a
Iscu
Ercc2
Slc25a5
Nubpl
Ezr
Msn
Slc9a3r1
Actb
L1cam
Icam1
Actg1
Slc9a1
Rhoa
Vcam1
RGD1304929
RGD1560436
Ugt3a2
Cwc25
Kb15
Slco1a5
Slco1a2
Slco1a1
Ilk
Tmsb4x
Actc1
Tbrg1
Gm17087

Brca1
Wdpcp
Cdk20
Ctdspl2
Qrich1
Bnip1
Suox
Gramd1c
Unc119
Crnkl1
Errfi1
Tubb5
Tubb1
Tubb6
Gzmb
Kcnq1
Mapre1
Grin1
Mtfp1
Cnnm3
Timmdc1
Traf6
Akt1
Sugt1
Cap2
Calm3
Polrmt
Msrb2
Rab11fip4
Trim21
Trim5
Trim17
Fibin
Slc22a5
Crtc2
Sod1
GSMUA_Achr10P22420_001
104000386
Slc25a39
Higd1b
Zfp523
Psmb1
Gpsm2
Rarres2
Gpha2
Gpr126
Nrbp1
Suv39h1

Smo
Heatr6
Rpa1
Ypel1
Slit1
Snph
Fam132a
Gnb3
Pax5
Mafk
Mafg
Mab21l1
Slc45a2
Mcoln1
Mcoln3
Slc25a19
Mcoln2
Lrrk2
Gp2
U2af2
Src
Ptk6
Ranbp3
Ddx3x
Siae
Fancl
Mdp1
Myh9
Mylk2
Aldob
Ube2d1
Mrpl2
Timm44
Txlng
Pccb
Map2k3
Narfl
Rdx
Lims1
Acta1
