# Supplementary material for: Systems Biology and Chemoinformatics-Based Strategies to Explore the Biological Mechanism of Fugui Wenyang Decoction in Treating Vascular Dementia Rats
Source: Oxid Med Cell Longev. 2021 Oct 7;2021:6693955. doi: 10.1155/2021/6693955 (PMC8517630; doi:10.1155/2021/6693955)
Supplement: Supplementary 6 — Table S6: bioinformatics analysis of differential expression of the protein-other rat protein PPI network. [file 6693955.f6.pdf]

Table S6 Bioinformatics Analysis of Differential expression protein-other rat protein PPI n

Category	Term	Descriptio	Count	%	PValue	Genes
	GO:0016226	iron-sulfur	9	2.093023256	6.14E-10	ISCA1, NI
	GO:0033173	calcineurin	6	1.395348837	1.22E-06	PPP3CA, I
	GO:0006096	glycolytic	8	1.860465116	2.12E-05	TPI1, PGK
	GO:0036120	cellular res	7	1.627906977	3.59E-05	ERRFI1, C
	GO:0060831	smoothene	4	0.930232558	3.58E-04	TBC1D32,
	GO:0021775	smoothene	3	0.697674419	0.001458457	SUFU, GL
	GO:0061028	establishm	4	0.930232558	0.004929044	RDX, MSI
Biological pro	GO:0051000	positive re	4	0.930232558	0.006949965	AKT1, CA
	GO:0032981	mitochond	4	0.930232558	0.00811872	NDUFS7,
	GO:0070527	platelet ag	5	1.162790698	0.012758824	ILK, MYH
	GO:0032543	mitochond	4	0.930232558	0.021506217	MRPL2, M
	GO:0071456	cellular res	8	1.860465116	0.033599178	BRIP1, SR
	GO:0070997	neuron dea	3	0.697674419	0.04279974	LRRK2, C
	GO:0014829	vascular sn	2	0.465116279	0.065254319	ACTA2, R
	rno04921	Oxytocin s	27	6.279069767	8.90E-13	MYLK2, F
	rno04022	cGMP-PK	27	6.279069767	4.10E-12	MYLK2, F
	rno04728	Dopaminer	21	4.88372093	2.61E-09	CALML3,
	rno04713	Circadian	16	3.720930233	2.83E-07	CALML3,
	rno04922	Glucagon s	16	3.720930233	4.26E-07	CRTC2, PI
	rno04611	Platelet act	18	4.186046512	9.30E-07	MYLK2, F
	rno04662	B cell rece	13	3.023255814	1.24E-06	CD72, NF
	rno04915	Estrogen s	15	3.488372093	1.48E-06	NOS3, SR
	rno04725	Cholinergi	16	3.720930233	1.88E-06	GNAI1, G
	rno04370	VEGF sign	12	2.790697674	2.03E-06	PPP3CA, I
	rno04724	Glutamater	16	3.720930233	2.65E-06	GNAI1, G
	rno04024	cAMP sign	21	4.88372093	2.75E-06	PPP1R12A
	rno04660	T cell rece	15	3.488372093	4.41E-06	IL10, NFA
	rno05014	Amyotroph	10	2.325581395	4.56E-05	MAP2K3,
	rno04071	Sphingolip	14	3.255813953	1.28E-04	ROCK2, N
Signaling path	rno04360	Axon guid	14	3.255813953	1.76E-04	ROCK2, N
	rno04020	Calcium si	17	3.953488372	2.17E-04	MYLK2, M
	rno04650	Natural kil	12	2.790697674	2.32E-04	PPP3CA, I
	rno04670	Leukocyte	13	3.023255814	2.85E-04	VCAM1, F
	rno04270	Vascular s	13	3.023255814	4.21E-04	MYLK2, F
	rno04015	Rap1 signa	18	4.186046512	4.25E-04	MAP2K3,
	rno05010	Alzheimer'	16	3.720930233	5.71E-04	NDUFA8,
	rno05214	Glioma	9	2.093023256	8.97E-04	AKT2, AK
	rno04120	Ubiquitin	13	3.023255814	0.001436918	UBE3C, F
	rno04727	GABAergi	10	2.325581395	0.001538547	GNAO1, C
	rno04722	Neurotroph	12	2.790697674	0.001943351	AKT2, TR
	rno04062	Chemokin	14	3.255813953	0.003670207	ROCK2, S
	rno04310	Wnt signal	12	2.790697674	0.004664415	PPP3CA, I
	rno04010	MAPK sig	17	3.953488372	0.006825934	MAP2K3,
	rno04726	Serotonerg	10	2.325581395	0.015709317	GNAO1, C
	rno04668	TNF signa	9	2.093023256	0.020875481	MAP2K3,

etwork

**Fold Enri**	**Bonferroni**
25.22618	1.41E-06
26.90793	0.00279
9.199292	0.047475
10.82503	0.078882
25.6266	0.559241
44.84655	0.964696
11.21164	0.999988
9.965899	1
9.441378	1
5.469091	1
6.643933	1
2.618776	1
8.969309	1
29.8977	1
5.641259	1.90E-10
5.293033	8.73E-10
5.169939	5.57E-07
5.238465	6.02E-05
5.081311	9.06E-05
4.234426	1.98E-04
5.983428	2.65E-04
4.962218	3.15E-04
4.536885	4.01E-04
6.351639	4.31E-04
4.418532	5.63E-04
3.420113	5.85E-04
4.536885	9.39E-04
5.774218	0.009661
3.585603	0.026859
3.473553	0.036895
2.918321	0.045194
3.888759	0.048119
3.528689	0.058965
3.38407	0.085738
2.646516	0.086555
2.791929	0.114587
4.397289	0.174015
2.948975	0.263822
3.650367	0.27961
3.02459	0.339221
2.511948	0.543055
2.702825	0.630589
2.100737	0.76751
2.561145	0.965701
2.622236	0.988819
