# Supplementary figures and images for: Systems Biology and Chemoinformatics-Based Strategies to Explore the Biological Mechanism of Fugui Wenyang Decoction in Treating Vascular Dementia Rats
Source: Oxid Med Cell Longev. 2021 Oct 7;2021:6693955. doi: 10.1155/2021/6693955 (PMC8517630; doi:10.1155/2021/6693955)

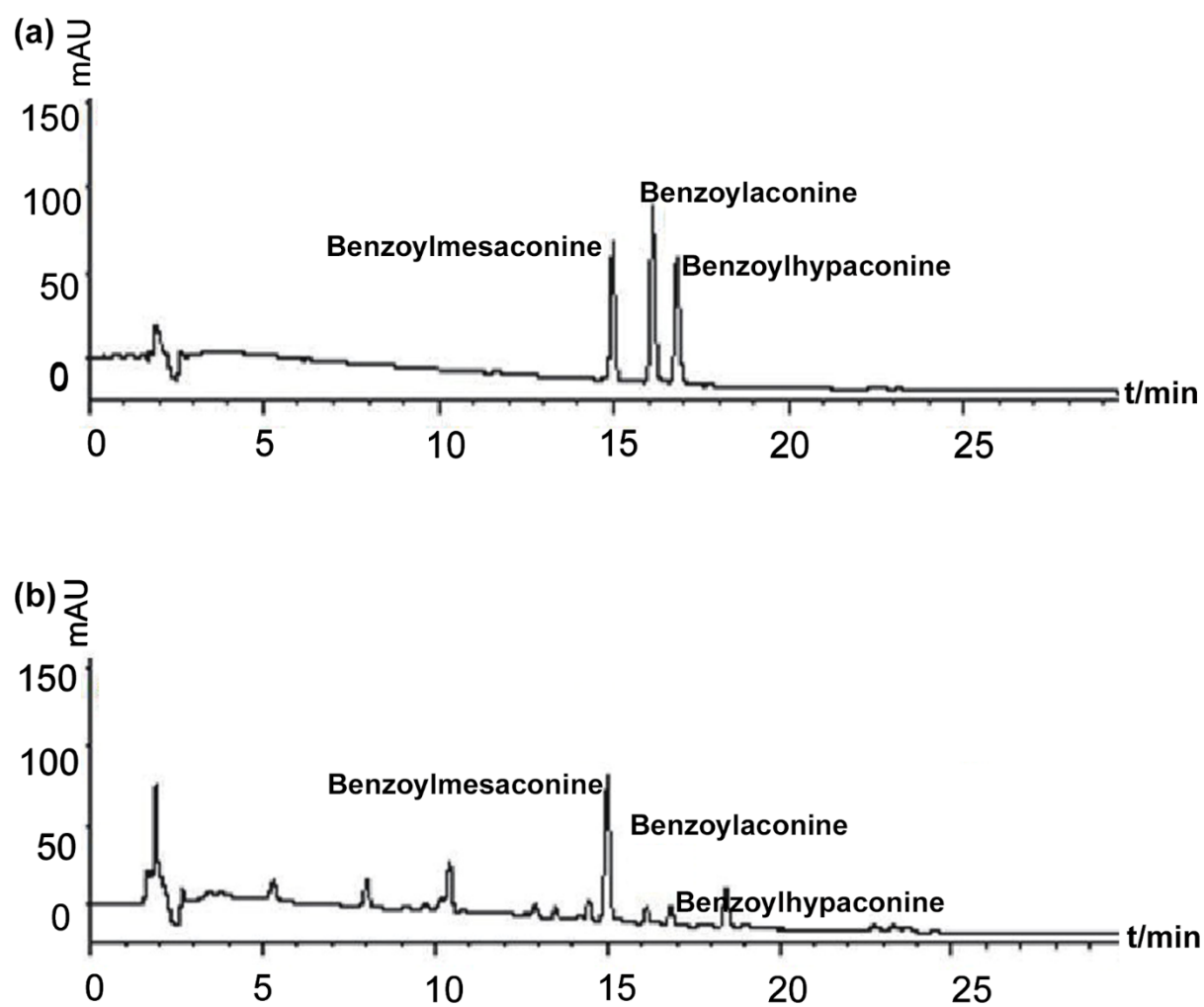

Figure S1 The results of HPLC (a: reference solution; b: FGWYD solution)

Supplement: Supplementary 7 — Figure S1: the results of HPLC: (a) reference solution; (b) FGWYD solution. [file 6693955.f7.pdf]
